# NOD/Scid IL2Rγ^null^ Mice Reconstituted with PBMCs from Patients with Atopic Dermatitis or Psoriasis Vulgaris Reflect the Respective Phenotype

**DOI:** 10.1016/j.xjidi.2024.100268

**Published:** 2024-02-03

**Authors:** Marietta Schindler, Paula Schuster-Winkelmann, Veronika Weß, Sophia Czell, Franziska Rueff, Andreas Wollenberg, Matthias Siebeck, Roswitha Gropp

**Affiliations:** 1 Department of General, Visceral, and Transplantation Surgery, LMU University Hospital, LMU Munich, Munich, Germany; 2 Department of Dermatology and Allergology, LMU University Hospital, LMU Munich, Munich, Germany; 3 Department of Dermatology and Allergology, University Hospital Augsburg, Augsburg, Germany

**Keywords:** Atopic dermatitis, Humanized mice, NOD/Scid IL2Rγ^null^ mice, PBMC, Psoriasis

## Abstract

NSG (NOD/Scid IL2Rγ^null^) mice reconstituted with PBMCs donated by patients with ulcerative colitis or Crohn’s disease highly reflect the respective pathological phenotype. To determine whether these findings could be applicable to atopic dermatitis (AD) and psoriasis vulgaris (PV), PBMCs isolated from patients with AD and PV were first subjected to immunological profiling. Subsequently, NSG mice were reconstituted with these PBMCs. Hierarchical clustering and network analysis revealed a distinct profile of patients with AD and PV with activated CD4+ T cells (CD69, CD25) occupying a central position in the AD network and CD4+ CD134+ cells acting as the main hub in the PV network. After dermal application of DMSO, both NSG mice reconstituted with PBMCs from donors with AD (ie, NSG-AD mice) and NSG mice reconstituted with PBMCs from donors with PV (ie, NSG-PV mice) exhibited increased clinical, skin, and histological scores. Immunohistochemical analysis, frequencies of splenic human leukocytes, and cytokine expression levels indicated that CD4+ CD69+ cells, M1 and TSLP receptor–expressing monocytes, switched B cells, and monocyte chemoattractant protein 3 were the driving factors of inflammation in NSG-AD mice. In contrast, inflammation in NSG-PV mice was characterized by an increase in fibroblasts in the epidermis, frequencies of CD1a-expressing monocytes, and IL-17 levels. Therefore, the pathological phenotypes of NSG-AD mice and NSG-PV mice differ and partially reflect the respective human diseases.

## Introduction

Narrowing the gap between manifestations of human diseases in patients and animal models remains a major challenge in the development and validation of novel therapeutics. This challenge is particularly significant in chronic inflammatory diseases, where the disease manifestations vary greatly in terms of disease onset, severity, duration of relapse and remission phases, and response to therapies. Conventional animal models for atopic dermatitis (AD) and psoriasis vulgaris (PV) typically involve the topical application of oxazolone or calcipotriol for AD (Liu et al, 2016; Tsukumo et al, 2010) and imiquimod for PV (Moos et al, 2019). Although these models have contributed to preclinical development, they have limited value in characterizing subtle differences that shape the immune response. Furthermore, preclinical studies are constrained to therapeutics that target both mouse and human molecular targets.

An alternative approach involves using immune-compromised mice (ie, NSG [NOD/Scid IL2Rγ^null^] mice) reconstituted with PBMCs from patients with the respective disease. This approach allows for the study of immunological specificities and the validation of therapeutics targeting human molecular targets. Previous studies have demonstrated that NSG mice reconstituted with PBMCs from patients with ulcerative colitis or Crohn’s disease reflect the pathophysiology of ulcerative colitis and Crohn’s disease, respectively (Unterweger et al, 2021b). These mice also partially capture the dynamics of inflammation observed in humans (Jodeleit et al, 2020) and have been instrumental in preclinical studies validating novel or approved therapeutics (Al-Amodi et al, 2018; Jodeleit et al, 2020, 2018; Unterweger et al, 2021a).

AD and PV are chronic inflammatory diseases that primarily affect the skin but exhibit distinct pathological manifestations underlying immunological processes (Guttman-Yassky and Krueger, 2017). AD results from a combination of skin barrier defects and an imbalanced immune system, leading to dry, itchy, and inflamed skin (Wollenberg et al, 2020). Dysfunction of the skin barrier can initiate the disease by allowing the penetration of allergens and microbes, triggering an immune response. However, dysregulated immune cells interacting with keratinocytes may also contribute to barrier defects, perpetuating inflammation. Initially, T helper (Th) 2–driven processes were considered the main drivers in AD, as evidenced by the therapeutic success of dupilumab (Simpson et al, 2016). However, activation of Th17 or Th1 pathways is also observed in many patients (Brunner et al, 2017; Noda et al, 2015; Suárez-Fariñas et al, 2013).

In contrast, psoriasis is clinically characterized by raised red scaly plaques caused by hyperproliferative keratinocytes, which are constantly exposed to infiltrating immune cells. It has been suggested that the influx of these cells is due to an autoimmune response in the skin (Arakawa et al, 2015; Lande et al, 2015, 2014). The IL-23/Th17 axis appears to be the main driver in these processes (Brembilla et al, 2018; Girolomoni et al, 2017). This observation has been supported by the successful treatment of patients with PV with anti–IL-17 mAbs such as secukinumab, bimekizumab, or ixelkizumab (Gordon et al, 2021, 2016; Krueger et al, 2019) as well as brodalumab, which inhibits the IL-17 receptor (McMichael et al, 2019).

In this study, we first examined the inflammatory profiles of patients with AD and PV and analyzed the respective immunological networks. The data indicate that both profiles were distinctly different. Secondly, we analyzed the pathological and phenotypic manifestation in NSG mice reconstituted with PBMCs from donors with AD or PV (ie, NSG-AD or NSG-PV, respectively). Our analyses suggest that the immunological background of the donor shapes the disease manifestations in NSG-AD and NSG-PV mice. Furthermore, the inflammatory profiles of NSG mice predominantly clustered according to the respective disease of the donors. These results suggest that the NSG-AD and NSG-PV models are well-suited for elucidating the immunological processes in AD and PV and for validating novel therapeutics.

## Results

### Comparison of immunological profiles of patients with AD and PV

**Figure 1:**
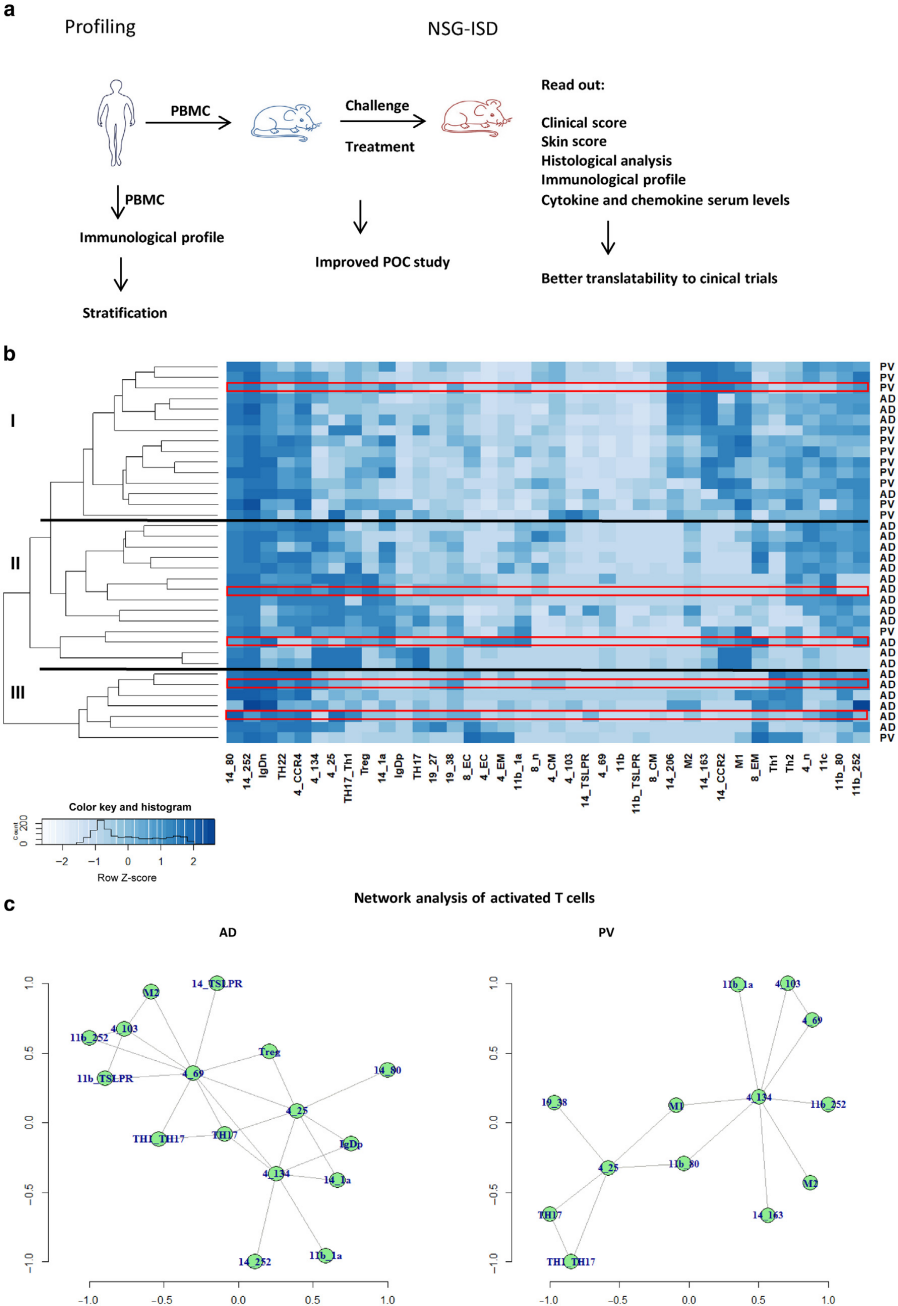
**The combination of animal studies in the NSG-AD and NSG-PV models has the potential to enhance our understanding of the underlying immunological processes.** (**a**) A schematic depiction of the experimental approach is presented. PBMCs were isolated from patients with AD or PV, subjected to immunological profiling, and then used for the reconstitution of NSG mice. (**b**) The analysis of patients with AD and PV was performed by hierarchical clustering. Frequencies of immune cells were determined through flow cytometric analysis of freshly isolated PBMCs from patients with AD (n = 23) and PV (n = 13). The profiles of 5 PBMCs from patients that were utilized for reconstitution in the animal model are highlighted in red. (**c**) Network analysis was conducted to explore significant correlations among surface markers of immune cells. AD, atopic dermatitis; PV, psoriasis vulgaris; Th, T helper; Treg, regulatory T cell.

The comparison of immunological profiles in patients with AD and PV followed a 2-pronged approach. First, we determined the immunological profile of patients, and second, the same PBMCs were used to reconstitute the NSG mice (Figure 1a). The immunological profiles of patients with AD (n = 23) and patients with PV (n = 13) were analyzed through hierarchical clustering analysis using the described panel (Table 1 presents the basic patient demographics, Table 2 presents the definition of cell types, and Table 3 shows the dataset of flow cytometry analysis of the patients).

**Table 1:** Basic Patient Demographics

Attributes	AD (N = 23)	PV (N = 13)
Age (y)		
Mean (SD)	23,857 (2,035)	28,25 (12,5)
Range	6	25
Sex (% male)	43%	25%
Duration of AD/PV (y)		
Mean (SD)	19,1429 (5,113)	4,75 (2,5)
Range	15	5
SCORAD/PASI		
Mean (SD)	14,323 (3,494)	7,925 (3,221)
Range	9,320	7,3
Treatment	Glucocorticoid topic (3), omalizumab (2), fexofenadine (2), salbutamol (2)	Mometasone (1), calcipotriol (1)

Abbreviations: AD, atopic dermatitis; PASI, Psoriasis Area and Severity Index; PV, psoriasis vulgaris; SCORAD, SCORing index of Atopic Dermatitis.

**Table 2:** Cellular Markers Used to Define Immune Cells

Marker	Definition	Abbreviation
CD19+ CD27+	Antigen experienced B cell	19_27
CD19+ CD27+ IgD+	Unswitched memory B cell	IgDp
CD19+ CD27+ IgD−	Switched memory B cell	IgDn
CD19+ CD38+	Plasma cell	19_38
CD19+ CD252+	Activated B cell	19_252
CD19+ CD38+ CD252+	Activated plasma cell	19_38_252
CD4+ CD45RA+ CD62L− CCR7−	Naïve CD4+ T-cell	4_n
CD4+ CD45RA+ CD62L+ CCR7+	Effector CD4+ T – cell	4_EC
CD4+ CD45RO+ CD62L− CCR7−	Effector memory CD4+ T cell	4_EM
CD4+ CD45RO+ CD62L+, CCR7+	Central memory CD4+ T cell	4_CM
CD4+ CD103+	Mucosal regulatory CD4+ T cell	4_103
CD4+ CCR4+	CCR4 expressing CD4+ T cell	4_CCR4
CD4+ CD25+ CD127−	Regulatory T cell	Treg
CD8+ CD45RA+ CD62L− CCR7−	Effector CD8+ T cell	8_EC
CD8+ CD45RA+ CD62L+ CCR7+	Naïve CD8+ T cell	8_n
CD8+ CD45RO+ CD62L− CCR7−	Effector memory CD8+ T cells	8_EM
CD8+ CD45RO+ CD62L+ CCR7+	Central memory CD8+ T cells	8_CM
CD4+ CCR4- CXCR3+ CCR6−	Th1	Th1
CD4+ CCR4- CCCR3+ CCR6+	Th1/Th17	Th1_Th17
CD4+ CCR4+ CCR6−	Th2	Th2
CD4+ CCR4+ CCR6+	Th17	Th17
CD4+ CCR4+ CCR6+ CXCR3− CCR10+	Th22	Th22
CD4+ CD134+	Activated CD4+ T cell	4_134
CD4+ CD69+	Activated CD4+ T cell	4_69
CD4+ CD25+	Activated CD4+ T cell	4_25
CD14+ TSLPR^1^+	MC, expressing TSLPR	14_TSLPR
CD14+ CD64+	M1 MC, FcγR1 expressing	M1
CD14+ CD163+ CD206+	M2 MC, scavenging cells	M2
CD14+ CD1a	MC CD1a expressing	14_1a
CD14+ CCR2+	MC activated	14_CCR2
CD14+ CD80+	MC activated	14_80
CD14+ CD252+	MC activated	14_252
CD14+ CD163+	MC expressing scavenger receptor	14_163
CD14+ CD206+	MC expressing mannose receptor	14_206
CD11b+	Macrophage	11b
CD11b+ CD80/86+	Macrophage activated	11b_80
CD11b+ TSLPR^1^+	macrophage TSLPR expressing	11b_TSLPR
CD11b+ CD1a+	Macrophage CD1a expressing	11b_1a
CD11b+ CD252+	Activated macrophage	11b_252
CD11c	Dendritic cell	11c

Abbreviations: MC, monocyte; Th, T helper; TSLPR, TSLP receptor.

**Table 3:** FACS Data Human PBMC

Disease	14_CCR2	M1	14_206	14_163	M2	14_1a	14_80	14_TSLPR	14_252	11b	11b_1a	11b_80	11b_TSLPR	11c	4_EC	4_n	4_CM	4_EM	8_n	8_EC	8_CM	8_EM	4_25	4_69	4_103	4_134	Treg	11b_252	19_27	IgDp	IgDn	19_38	4_CCR4	Th2	Th17	Th22	Th1	Th17_Th1
AD	52.20	74.20	4.48	61.30	7.02	68.90	55.70	0.31	73.10	1.09	81.90	8.98	0.70	11.70	74.40	3.16	2.11	72.30	0.00	61.50	0.00	92.30	12.90	1.22	1.95	49.00	49.00	73.40	21.20	6.07	93.30	28.70	7.80	41.30	22.10	10.50	23.30	57.10
AD	100.00	97.50	0.00	25.00	0.00	47.70	92.00	0.26	99.60	0.00	0.00	0.00	0.00	9.60	16.50	35.90	0.00	0.00	0.00	0.00	9.69	30.40	99.90	17.90	0.69	99.70	8.82	0.00	25.80	78.60	18.70	42.30	10.90	0.11	99.70	32.00	1.06	98.40
AD	100.00	94.30	0.00	37.70	3.77	14.60	96.50	0.13	99.80	0.00	0.00	0.00	0.00	4.08	0.00	25.00	0.00	0.00	0.00	0.00	13.60	15.40	100.00	13.50	0.27	99.90	9.46	0.00	24.80	62.60	34.40	39.50	37.00	2.00	96.30	55.00	2.96	96.10
AD	0.00	0.00	50.00	0.00	50.00	29.80	25.40	1.16	99.60	0.25	13.00	26.60	4.55	22.80	0.00	0.00	12.80	27.70	0.00	0.00	4.20	65.00	12.00	14.30	24.70	42.00	2.04	94.80	10.60	1.65	88.10	25.00	30.80	64.20	26.10	40.80	54.30	34.70
AD	0.00	0.00	0.00	0.00	0.00	4.63	90.30	0.41	95.70	7.63	6.29	68.50	0.06	57.10	0.10	16.60	31.20	11.20	27.90	6.21	5.12	7.65	30.20	3.09	1.15	54.10	10.60	73.20	33.70	1.48	97.70	61.80	85.00	74.70	6.90	66.40	53.50	30.70
AD	13.30	11.80	0.03	0.12	25.10	92.00	94.30	0.22	99.70	0.12	58.10	72.60	0.00	85.10	0.00	74.80	16.70	50.00	31.20	6.25	0.00	85.20	81.80	3.64	1.10	97.90	10.60	77.40	34.50	16.80	78.00	56.60	56.10	84.70	10.00	23.80	56.00	40.00
AD	15.80	18.10	0.19	0.19	50.50	94.30	92.30	11.70	99.80	0.06	82.60	69.60	21.70	86.20	18.80	12.50	10.00	20.00	0.00	0.00	5.15	30.00	99.90	50.00	22.00	99.80	98.90	91.30	48.40	47.60	42.60	81.70	94.20	1.59	97.90	91.90	0.00	71.90
AD	5.32	37.20	0.39	2.37	34.90	9.12	90.60	1.95	99.00	4.66	38.20	69.40	1.90	66.50	0.00	91.40	59.50	6.33	91.70	0.00	3.70	59.30	45.20	7.72	11.50	87.60	12.30	73.10	49.40	1.87	97.00	41.70	86.80	48.50	16.10	77.20	38.40	37.20
AD	0.21	0.41	0.03	0.04	0.98	29.00	96.70	4v04	97.00	3.00	19.80	27.00	0.20	7.90	41.00	3.57	16.20	22.10	0.00	88.90	1.59	79.00	73.80	6.80	0.53	76.40	4.70	67.60	83.20	11.40	87.90	31.70	95.60	8.54	60.60	91.80	0.00	0.00
AD	0.72	5.32	0.86	1.29	48.10	68.10	89.40	2.21	98.90	0.67	43.70	60.20	1.06	82.50	0.00	98.60	43.80	18.80	42.90	21.40	0.00	55.60	81.30	9.55	5.45	98.40	20.80	86.60	15.30	9.40	89.50	19.50	99.60	71.10	18.70	99.00	0.00	0.00
AD	2.38	1.19	0.00	1.19	11.90	89.90	98.30	0.40	99.90	0.01	40.00	100.00	0.00	85.30	1.69	70.70	0.00	0.00	0.00	0.00	4.94	29.60	98.90	8.52	7.23	98.50	95.90	80.00	26.60	33.40	63.80	31.70	99.60	77.20	19.50	96.70	0.00	0.00
AD	1.28	3.85	1.28	2.14	49.60	81.50	95.60	1.05	99.40	0.86	47.70	55.90	0.20	75.30	5.19	62.20	25.00	25.00	0.00	0.00	0.00	100.00	46.10	14.70	7.66	59.10	20.20	82.50	18.10	10.60	87.70	32.10	99.50	72.10	25.70	98.70	0.00	66.70
AD	2.59	3.14	0.20	1.89	18v00	60.20	93.80	0.47	92.60	3.22	22.70	62.70	0.05	69.80	0.63	84.50	11.40	70.50	70.50	17.20	5.26	89.50	18.70	1.34	0.64	52.20	15.20	67.70	18.70	0.80	99.00	36.60	97.70	67.70	20.90	89.80	0.00	100.00
AD	3.21	2.70	0.00	2.53	15.40	5.07	96.30	0.00	98.80	1.45	0.73	51v80	0.00	72.10	0.71	44.40	8.87	32.30	0.00	50.00	0.00	0.00	13.20	0.90	0.31	32.70	11.50	72.50	12.60	0.83	99.10	34.50	99.90	83.30	14.90	99.40	100.00	0.00
AD	14.20	38.50	26.80	17.10	84.90	81.00	99.50	86.80	99.40	0.46	33.40	86.00	3.75	73.40	3.10	80.50	75.00	16.70	0.00	0.00	31.30	24.70	99.50	50.30	13.70	93.50	98.70	57.00	22.50	70.50	26.60	27.80	89.90	10.00	77.10	79.10	5.30	91.70
AD	4.31	0.43	0.09	0.26	17.20	59.00	99.30	0.44	99.50	0.00	0.00	0.00	0.00	90.90	23.80	33.00	0.00	0.00	24.70	34.20	0v00	0.00	99.50	5.64	0.66	67.10	98.60	0.00	33.40	10.50	86.50	33.90	88.30	38.40	43.90	78.10	11.90	83.70
AD	3.61	0.52	0.00	0.52	9.79	42.90	95.90	0.29	99.40	0.00	0.00	0.00	0.00	92.30	19.80	42.20	0.00	0.00	29.30	30.80	0.00	0.00	99.90	47.10	1.05	95.70	97.60	0.00	13.70	25.00	68.70	28.70	70.60	84.90	13.40	70.30	4.41	89.80
AD	12.20	11.70	2.17	5.57	30.10	28.40	99.80	43.80	62.50	1.55	7.25	83v50	4.13	64.90	11.60	39.40	16.70	21.10	18.20	45.50	13.80	37.10	92.50	1.44	0.91	1.12	30.00	36.90	36.00	14.20	84.40	49.00	87.70	17.90	42.00	76.90	21.70	74.50
AD	42.50	85.30	0.24	57.10	0.38	77.80	79.70	2.09	81.70	6.08	73.30	68.00	3.26	45.20	0.00	2.44	0.00	50.00	0.00	14.30	0.00	0.00	50.90	20.40	10.30	48.00	52.10	39.20	14.50	46.60	48.00	53.70	79.30	51.10	28.80	47.30	20.00	47.70
AD	97.00	90.70	0.96	94.50	3.20	15.40	83.20	0.23	100.00	11.40	37.30	86.50	19.30	65.30	8.14	64.10	45.00	10.60	60.60	7.77	30.70	18.90	31.50	1.09	1.20	38.10	36.80	85.90	11.50	3.47	95.20	24.90	78.80	50.10	22.50	67.00	32.90	54.90
AD	96.70	79.80	89.40	96.80	84.20	79.00	87.00	0.64	96.40	11.00	54.40	59.10	13.30	26.30	20.50	24.70	18.10	36.10	16.10	18.40	16.70	45.80	43.10	9.74	7.44	73.90	43.50	79.80	4.93	40.40	57.60	64.40	70v10	33.90	24.50	32.20	11.80	58.80
AD	0.00	63.60	13.60	9.09	13.60	0.24	93.20	1.65	96.50	0.18	0.00	64.00	4.00	0.01	0.00	0.00	0.00	0.00	0.00	0.00	1.12	61.20	33.30	0.00	0.00	0.00	33.30	0.00	0.09	0.00	87.50	24.60	84.10	65.40	7.38	71.10	70.30	15.30
AD	81.10	67.40	41.40	0.54	19.50	2.47	79.50	47.40	99.00	2.04	0.00	55.40	20.00	68.20	0.32	45.60	28.20	16.50	8.21	26.10	5.94	49.50	18v00	0.34	8.11	43.40	33.90	74.60	13.00	0.67	99.20	20.80	92.70	61.10	19.70	92.00	57.70	31.50
PV	41.00	84.20	80.10	90.90	74.50	26.00	90.40	25.30	98.40	11.20	10.20	64.50	0.12	49.50	7.12	51.10	34.20	12.50	0.00	27.30	12.80	46.20	34.60	16.50	17.30	15.50	32.10	69.70	30.30	24.30	71.80	41.80	87.40	50.10	22.40	54.30	33.70	36.10
PV	51.20	87.00	82.90	91.30	76.00	66.40	89.80	2.43	99.20	7.68	13.90	57.40	0.21	63.80	10.20	42.00	29.70	23.20	15,20	19.70	12.10	42.40	13.10	9.36	1.71	47.30	26.90	51.80	26.80	20.80	76.40	36.30	83.00	58.70	34.50	64.10	14.90	68.10
PV	0.00	33.30	0.00	0.00	0.00	1.14	89.10	7.20	98.90	0.21	0.00	46.20	12.80	0.01	84.20	0.99	0.00	90.20	0.00	100.00	0.00	100.00	0.96	0.02	8.46	0.00	3.69	17.90	1.37	3.37	96.30	8.62	81.10	80.00	20.00	50.00	75.00	0.00
PV	86.30	95.30	40.40	75.70	25.50	85.60	86.10	16.40	99.40	11.60	33.10	48.20	0.26	46.60	22.40	38.70	17.70	23.30	25.60	45.50	12.90	41.90	48.70	4.82	2.04	42.20	55.10	50.10	23.00	15.20	81.70	48.50	83.40	48.90	25.90	90.80	53.60	22.30
PV	90.20	96.10	49.00	58.80	33.30	51.90	90.70	16.80	99.10	9.65	52.30	46.20	0.46	57.70	1.02	90.50	41.60	18.00	27.00	53.40	16.70	47.20	30.70	0.09	0.67	7.31	37.60	37.40	26.50	9.79	89.30	21.50	84.20	65.30	16.40	88.80	52.20	37.00
PV	91.10	90.90	74.20	85.80	64.90	6.41	88.80	15.90	99.70	3.01	1.77	72.00	1.28	72.50	3.99	59.50	20.40	19.70	36.80	14.80	20.60	14.30	98.70	4.15	2.90	6.21	19.40	78.10	23.20	16.70	78.20	45.80	70.40	11.30	84.50	58,10	3.15	95.90
PV	8.18	78.50	96.50	97.70	92.50	12.50	89.10	14.90	99.60	3.64	16.90	72.40	3.37	68.70	1.99	50.20	40.40	11.00	17.00	38.80	25.00	29.20	40.70	15.50	13.50	55.50	47.10	73.40	23.30	4.53	94.40	35.00	84.80	55.70	20.70	63.20	47.00	37.80
PV	87.00	91.60	81.40	95.50	74.80	20.70	80.00	17.50	99.00	11.20	4.28	61.60	3.70	47.00	0.16	71.10	47.60	9.92	46.30	6.69	29.30	12.20	31.80	19.50	19.10	62.60	33.20	93.90	22.00	11.20	65.90	21.00	77.40	46.60	24.40	61.30	31.00	50.70
PV	89.60	72.60	99.40	99.50	99.10	81.00	98.00	26.00	99.00	3.93	5.64	69.10	21.90	66.00	0.24	77.20	53.30	9.53	41.00	25.80	25.70	29.40	27.80	21.20	21.80	22.30	37.90	63.90	45.40	4.21	56.20	28.40	58.50	38.60	24.40	30.20	25.50	51.90
PV	59.40	40.60	65.60	84.40	51.60	80.40	95.10	1.27	97.90	1.81	0.00	41.80	0.12	64.40	0.36	34.90	25.40	8.75	25.00	27.50	19.00	29.50	19.30	2.84	2.49	8.43	52.30	61.30	9.31	5.44	92.60	8.14	74.40	69.10	22.20	64.20	37.40	56.40
PV	85.90	38.90	60.60	94.60	38.90	58.20	77.70	1.54	99.20	0.78	0.23	58.50	0.46	76.00	5.78	51.80	25.80	27.60	15.50	43.90	29.30	31.70	21.80	4.55	1.28	9.22	56.60	80.00	4.29	1.50	95.90	14.80	63.50	71.60	19.20	46.80	59.20	30.30
PV	21.40	71.40	53.60	32.10	32.10	72.80	86.90	70.40	99.80	7.27	6.28	68.70	3.93	60.50	6.13	42.80	75.00	0.00	0.00	0.00	0.00	0.00	98.60	2.87	95.70	2.40	2.44	77.20	24.90	21.30	75.10	34.00	63.00	40.60	54.30	17.70	46.00	43.00
PV	41.70	85.40	37.50	41.70	20.80	50.50	87.10	25.30	99.80	0.88	0.00	46.10	38.30	3.83	0.58	44.10	35.70	14.30	28.00	11.80	26.20	41.50	33.60	20.50	0.72	6.04	52.90	63.00	24.60	45.80	51.20	19.00	84.80	74.70	9.10	48.10	23.80	57.10

Abbreviations: AD, atopic dermatitis; PV, psoriasis vulgaris; Th, T helper.

**Figure 2:**
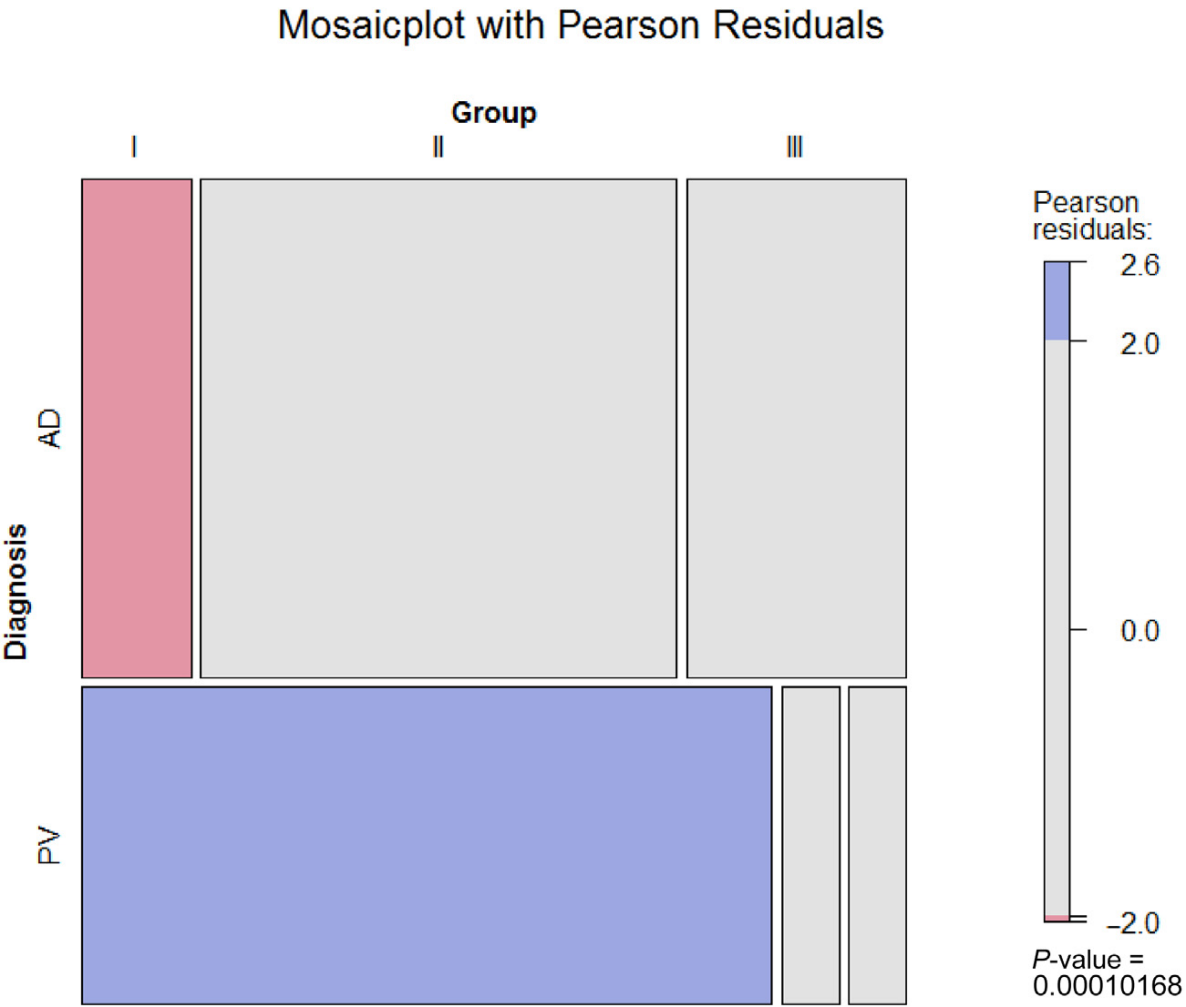
**Mosaic plot and Pearson residual contingency analysis of immunological profiles in humans.** The width of the rectangle indicates the number of samples. Red tiles indicate significant negative residuals, where the frequency is less than expected. Blue tiles indicate significant positive results, where the frequency is greater than expected. Labels on the right side indicate the contribution of each cellular profile to the significance of the chi-squared test result.

The heat map (Figure 1b) indicates that patients with AD and PV generally segregate into different groups on the basis of the peripheral blood inflammatory profiles and revealed 3 main groups. Group I exhibited significantly elevated frequencies of CD4+ and CD8+ central memory T cells, macrophages, and activated macrophages. Notably, the most prominent difference was observed in the analysis of monocytes, where most patients with PV (12 of 14) clustered in this group compared with only 3 of 22 patients with AD. Group II differed significantly from the other 2 groups owing to elevated frequencies of activated CD4+ cells, Th17/Th1 cells, regulatory T cells, and CD1a-expressing monocytes and macrophages. This group mainly consisted of patients with AD (13 of 22) and 1 patient with PV. In group III, significantly elevated frequencies of Th1 and switched B cells were observed, along with reduced levels of naïve CD4+ and dendritic cells. This group included 6 of 22 patients with AD and 1 patient with PV, indicating a subgroup of patients with AD driven by a Th1 response. To confirm the significance of the clustering, the data were visualized using a mosaic plot and analyzed through Pearson residual contingency testing (Figure 2). The results indicate that the clustering of patients with PV in group I is significant and that inflammation in PV and AD is driven by different cell types.

To further understand the immunological equilibria in AD and PV, a network analysis based on significant correlations of cell surface marker expression was conducted. The lines between individual dots in the network represent correlations, whereas the distance illustrates the strength of the correlation. In the network of AD (Figure 1c), dots were more widely distributed than the PV network. Several hubs were identified, centered on Th1/Th17 and Th17 cells, activated CD4+ CD69+ cells, regulatory T cells, and CD14+ TSLP receptor–positive monocytes. In contrast, the PV network appeared denser, with 1 main hub centered on activated CD11b+ CD252+ macrophages. In both networks, activated CD14+ CCR2+ monocytes correlated with Th1/Th17 and Th17 cells.

### Comparison of the NSG-AD with NSG-PV mouse models

On the basis of previous studies that showed partial preservation of the immunological phenotype of the donor in the NSG mouse model (Jodeleit et al, 2020; Unterweger et al, 2021b), we anticipated the development of distinct phenotypes in mice reconstituted with PBMCs from patients with AD compared with those from mice reconstituted with PBMCs from patients with PV. NSG mice were reconstituted with PBMCs from donors with AD (NSG-AD) or PV (NSG-PV). The selected immune profiles of 5 donors with AD and PV for the animal studies are highlighted in Figure 1b. Considering previous findings with the NSG-ulcerative colitis and NSG-Crohn’s disease model, where mild toxins such as ethanol were sufficient to induce disease symptoms, we utilized DMSO assuming that its skin penetration and exposure to skin pathogens might likewise induce similar symptoms. NSG mice reconstituted with PBMCs from a healthy donor served as control.

On the eighth day after reconstitution, the mice were depilated and divided into 2 groups: one group remained unsensitized (control group), whereas the other was sensitized through topical application of DMSO (DMSO group). Table 4 presents the group composition details.

**Table 4:** Animals and Groups Defined in the Animal Studies

Donor	Diagnosis	Medication	SCORAD/PASI	Groups in the NSG Model	
				Control, n (f/m)	DMSO, n (f/m)^1^
AD1	AD	None	17.36	4 (2/2)(0)	8 (6/2)(1)
AD2	AD	Glucocorticoid topic	12.3	6 (2/4)(0)	6 (6/0)(0)
AD3	AD	Omalizumab, glucocorticoid topic, fexofenadin, salbutamol	12.608	0	6 (6/0)(1)
AD4	AD	Omalizumab, glucocorticoid topic, fexofenadine, salbutamol	15.054	0	6 (4/2)(0)
AD5	AD	None	17.36	0	6 (6/0)(0)
AD6	AD	Glucocorticoid topic	17.5	0	6 (6 /0)(0)
AD7	AD	None	8.18	0	6 (4/2)(2)
PV1	PV	None	8.7	6 (0/6)(0)	6 (6/0)(0)
PV2	PV	None	4.8	6 (4/2)(0)	6 (0/6)(0)
PV3	PV	None	6.1	0	6 (3/3)(0)
PV4	PV	Mometasone, calcipotriol	12.1	0	6 (6/0)(0)
H	Healthy		0	6 (6/0)(0)	6 (6/0)(0)
Total				28 (14/14)(0)	74 (59/15)(4)

Abbreviations: AD, atopic dermatitis; f, female; m, male; PASI, Psoriasis Area and Severity Index; PV, psoriasis vulgaris; SCORAD, SCORing index of Atopic Dermatitis.

1 Animals excluded from the study.

**Figure 3:**
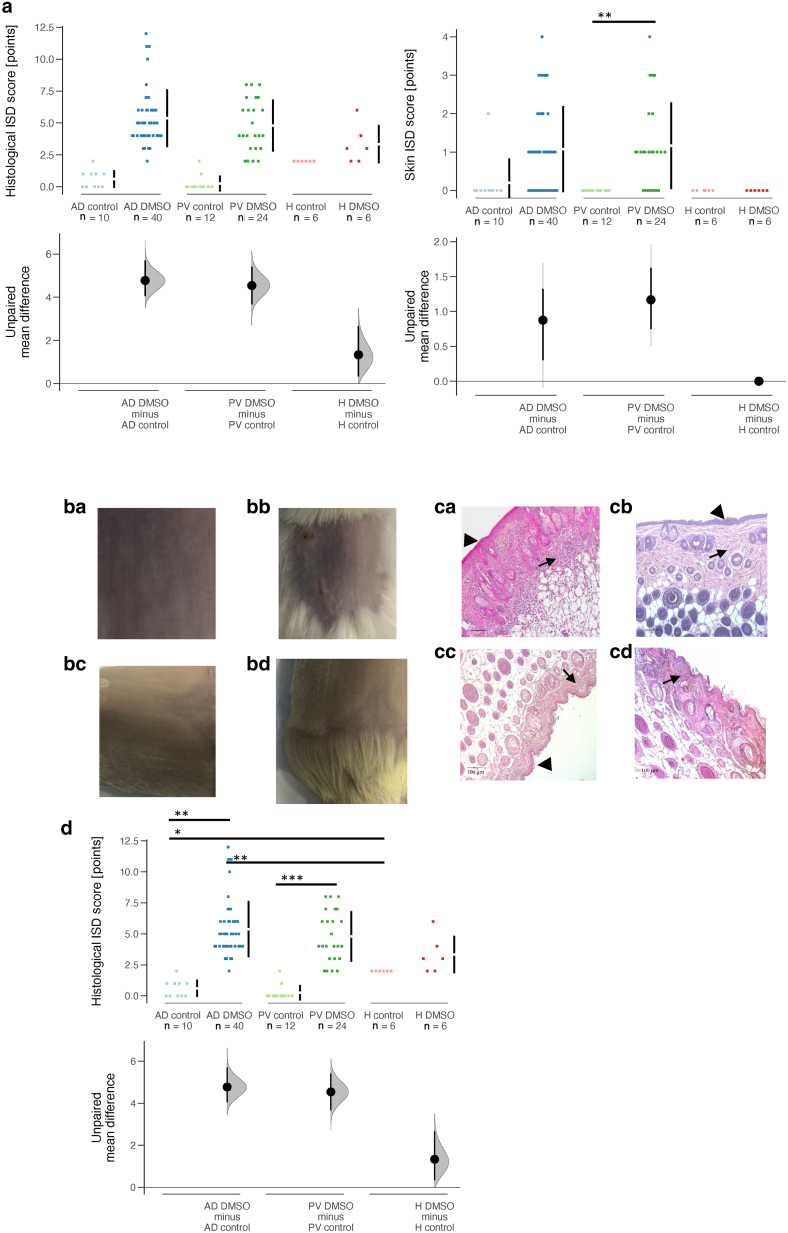
**Exposure to DMSO induces skin lesions and pathological manifestations in NSG-AD and NSG-PV mice.** NSG mice were reconstituted with PBMCs from donors with AD (n = 7) and PV (n = 4) or healthy donors (n = 1) on day 1 or left nonreconstituted. H denotes healthy. They were either left unchallenged (AD control: n = 10; PV control: n = 12; healthy control: n = 6) or challenged with 100% DMSO on days 8, 16, 18, and 20 (AD DMSO: n = 43; PV DMSO: n = 24; healthy DMSO: n = 6). The mice were killed on day 21. (**a**) Clinical ISD scores and skin ISD scores are presented as Cumming plots. (**b**) Representative macrophotographs of the skin of (**a**) AD DMSO, (**b**) PV DMSO, (**c**) healthy DMSO, and (**d**) nonreconstituted DMSO mice are shown. (**c**) Representative micrographs of H&E-stained skin sections from (**a**) AD DMSO, (**b**) PV DMSO, (**c**) healthy DMSO, and (**d**) nonreconstituted DMSO mice. Bar = 100 μm. Arrows indicate the influx of inflammatory cells, whereas bold arrows indicate the thickening of the skin. (**d**) Histological ISD scores are depicted as a Cumming plots. ∗*P* = .05–.01, ∗∗*P* = .01–.001, and ∗∗∗*P* < .001. AD, atopic dermatitis; ISD, inflammatory skin disease; PV, psoriasis vulgaris.

As expected, mice in the AD and PV DMSO groups exhibited symptoms on the skin, such as redness or bloody spots. In contrast, the skin of NSG-H mice exhibited almost no signs of irritation. These symptoms were categorized as part of a clinical inflammatory skin disease score, as described in the Materials and Methods (Figure 3a). The clinical inflammatory skin disease score showed a significant difference when comparing the AD and PV control groups with the respective DMSO-challenged groups (AD control vs AD DMSO: *P* = .02, PV control vs PV DMSO: *P* = .009; ANOVA). However, there was no significant difference observed between the AD DMSO and PV DMSO groups and between the healthy control and healthy DMSO groups.

At the time of necropsy, photographs of the skin were taken (Figure 3b), and skin symptoms were scored as described in the Materials and Methods (Figure 3a). Consistent with the clinical score, there were differences in the skin scores between the respective control and DMSO-challenged groups (AD control vs AD DMSO: *P* = .01; PV control vs PV DMSO: *P* < .001, Wilcoxon rank sum test with continuity correction) with the exception of the healthy control). However, there was no significant difference in scores between the AD DMSO and PV DMSO groups.

Histological sections of the skin were stained with H&E, and alterations in skin architecture were classified using a histological inflammatory skin disease score, as outlined in Materials and Methods (Figure 3c).

Both models showed an influx of inflammatory cells into the skin and thickening of the skin.

In the NSG-AD and NSG-PV models, the sensitized groups had noticeably higher scores than the respective control groups (AD control vs AD DMSO: *P* = not significant; PV control vs PV DMSO: *P* = not significant, ANOVA), whereas the difference between healthy control and healthy DMSO was not significant.

**Figure 4:**
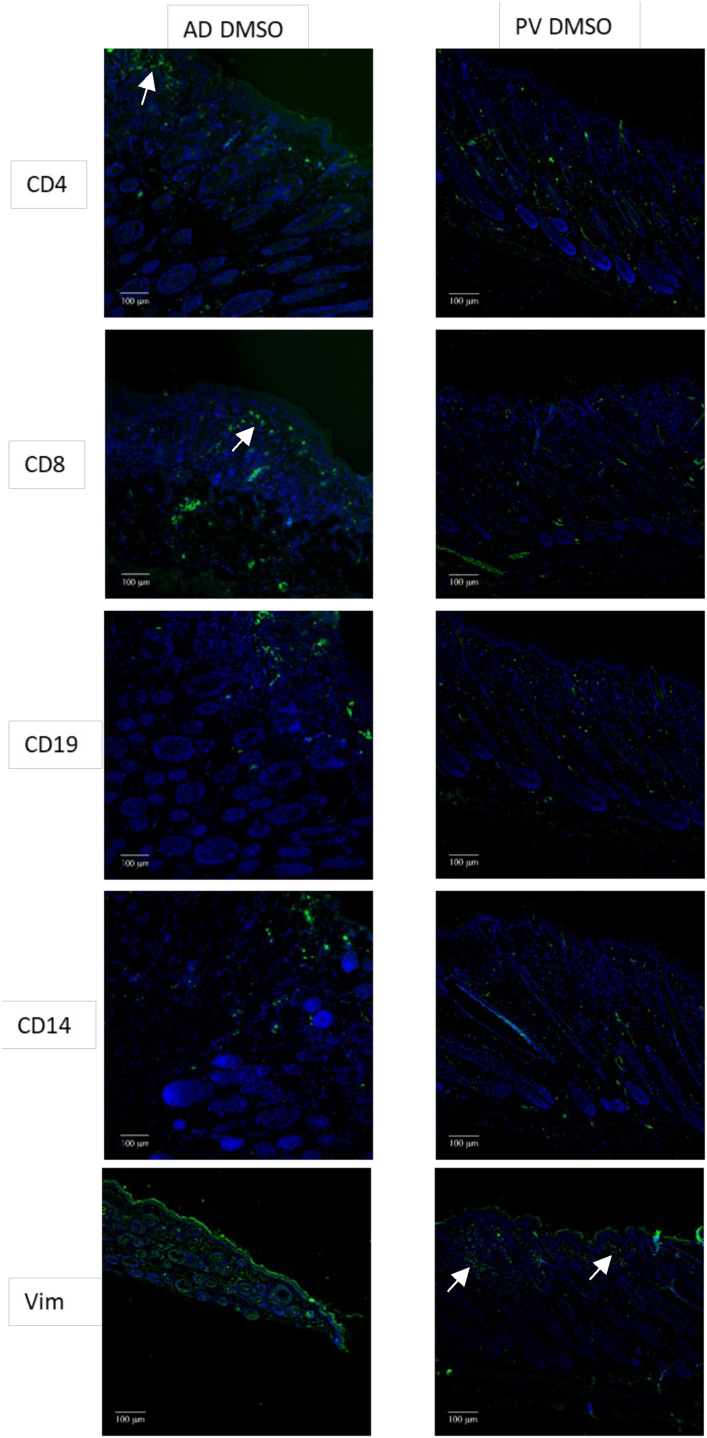
**Illustration of the influx of different immune cells into the epidermis of DMSO-treated NSG-AD and NSG-PV mice.** The mice were treated as described in the previous section. Sections were stained with anti-human CD4, anti-human CD8, anti-human CD19, anti-human CD14, and anti-mouse Vim antibodies. Micrographs of representative sections were captured using an Axioskop 40 CFL camera (Zeiss). To enhance contrasts within the images, a tonal correction was applied using Adobe Photoshop CC. Bar = 100 μm. AD, atopic dermatitis; PV, psoriasis vulgaris; Vim, vimentin.

To characterize the human immune cells in the dermis, immunohistochemical analysis was conducted using anti-human CD4, CD8, CD19, and CD14 and anti-mouse vimentin antibodies. In NSG-AD mice, the challenge with DMSO seemed to have led to a more pronounced influx of CD4+ and CD8+ T cells, CD14+ monocytes, and CD19+ B cells than challenged NSG-PV mice (Figure 4). In contrast, NSG-PV mice seemed to exhibit more detectable positive vimentin, indicating increased fibrosis in this model.

**Figure 5:**
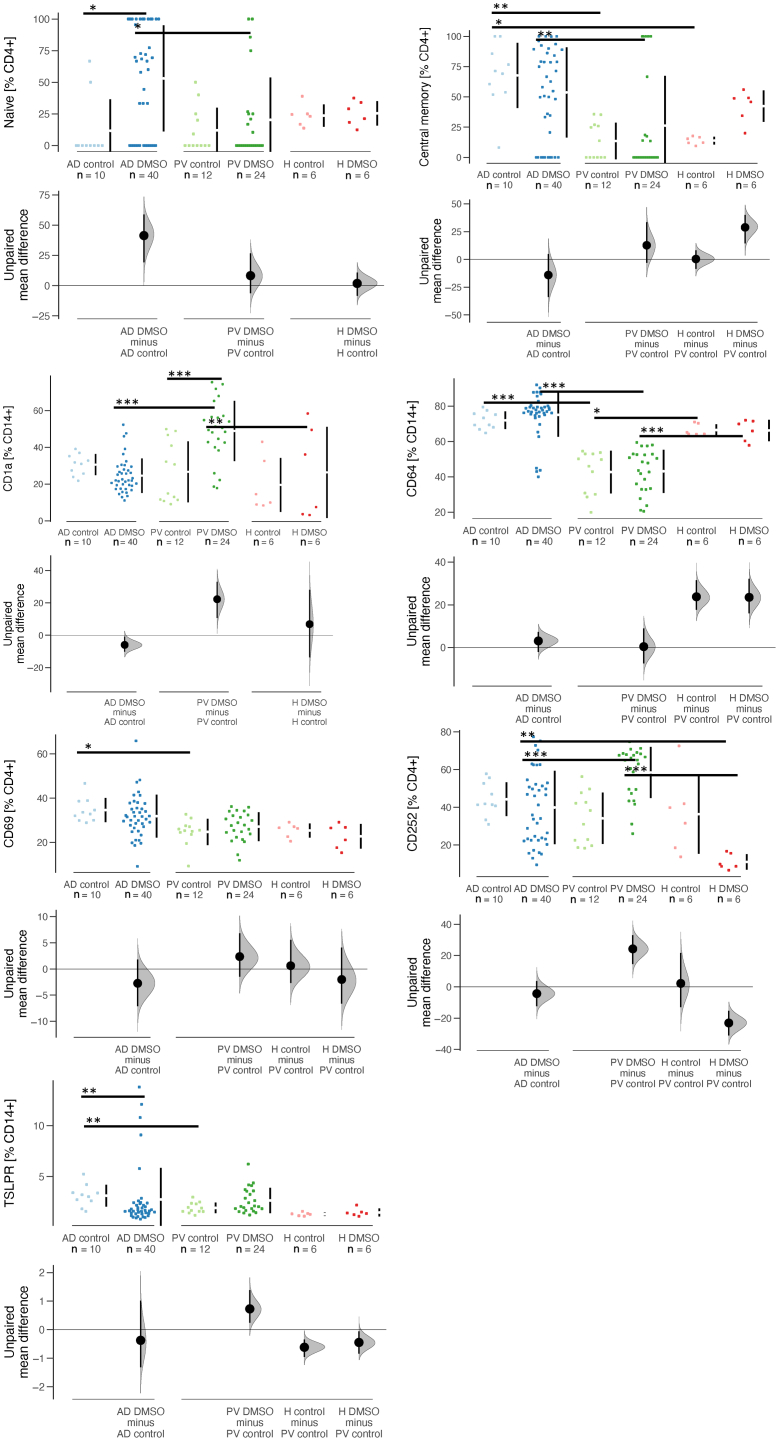
**Display of the distinct immunological phenotypes of NSG-AD and NSG-PV mice.** The mice were treated as described in the previous section, and human leucocytes were isolated from their spleens for flow cytometric analysis. Frequencies of different cell types are presented as Cumming plots. ∗*P* = .05– .01, ∗∗*P* = .01– .001, and ∗∗∗*P* < .001. AD, atopic dermatitis; PV, psoriasis vulgaris.

Leukocytes isolated from the spleen of mice were subjected to flow cytometry analysis (Figure 5). Levels of naïve CD4+ were significantly elevated in sensitized NSG-AD versus NSG-PV mice (*P* = .01, ANOVA) and in the NSG-AD and NSG-PV models; higher levels of naïve CD4+ T cells were observed upon sensitization with DMSO (AD control vs AD DMSO: *P* = .02; PV control vs PV DMSO: *P* = not significant, ANOVA). Levels of central memory CD4+ T cells did not change in response to DMSO in the NSG-AD and NSG-PV models; however, levels increased in the NSG-H model, albeit without significance. Interestingly, NSG-AD mice exhibited significantly higher levels of central memory CD4+ T cells than the respective groups of NSG-PV and NSG-H mice (AD control vs PV control: *P* = .004; AD DMSO vs PV DMSO: *P* = .007, healthy control vs AD control: *P* = .03, ANOVA).

Levels of CD4+CD69+ activated T cells did not change upon sensitization with DMSO; however, levels were higher in NSG-AD mice than in NSG-PV mice. The difference between the control groups (*P* = .03, ANOVA) was significant. Levels of activated CD4+ T cells characterized by CD252 expression responded to sensitization with DMSO significantly in NSG-PV mice (PV control vs PV DMSO: *P* = .001, ANOVA). In addition, a significant difference was observed between AD DMSO and PV DMSO (*P* < .001) and between PV DMSO and healthy DMSO (*P* < .001) and H DMSO and AD DMSO (*P* = .006, ANOVA).

Similarly, CD14+ CD1a+ monocytes responded to the challenge with DMSO in NSG-PV mice (PV control vs PV DMSO: *P* < .001, ANOVA). The difference between the AD DMSO and PV DMSO groups (*P* = not significant) and between PV DMSO and healthy DMSO was also significant (*P* = .005, ANOVA).

In none of the models, sensitization affected the levels of M1 monocytes (CD14+ CD64+). However, a striking difference was observed between the NSG-AD and NSG-PV and NSG-H models regardless of the challenge. The NSG-AD mice exhibited elevated levels of M1 monocytes compared with those of the other models (AD control vs PV control: *P* < .001; AD DMSO vs PV DMSO: *P* = not significant, healthy control vs PV control: *P* = .001, healthy DMSO vs PV DMSO: *P* < .001, ANOVA). Frequencies of CD14+ TSLP receptor–positive expressing monocytes responded to DMSO sensitization in NSG-AD mice, and a significant difference was observed between the respective control groups (AD control vs AD DMSO: *P* = .005; AD control vs PV control: *P* = .004; AD DMSO vs PV DMSO: *P* = .09, Wilcoxon rank sum test with continuity correction). Table 5 presents the dataset of flow cytometry analysis of mice.

**Table 5:** Data Mice

Donor	Sex	Treatment	Clinical ISD Score	Histological ISD Score	Skin ISD Score	19	19_38	27n	27n_IgDn	27n_IgDp	27p	27p_IgDn	27p_IgDp	4_n	4_EC	4_CM	4_EM	14_1a	14_252	14_TSLPR	14_163	14_206	M1	M2	4_69	4_103	4_134	Treg	4_252	MCP3	TSLP	IL-4	IL-12p70	IL-17A
AD 1	m	AD control	0	0	0	41.8	5.67	81.4	48.3	48	15.6	54.3	41.6	0	11.1	8.19	59.8	23.9	12.4	1.57	75.6	28.1	75.2	31.8	32	22	4.66	0.84	40.7	910	114	77	28	28
AD 1	m	AD control	0	0	0	45.7	5.01	82.1	39.6	57.3	13.9	38.3	56.7	0	0	60.6	15.5	37	18.5	2.72	84.4	31	75.7	35.4	32.8	22.6	5.43	0.23	46.9	2019	54	76	27,5	28
AD 1	f	AD control	0	0	0	59.8	14.6	74.4	31.7	63.3	20.6	24.2	72.1	0	0	76.7	4.11	39.1	15.9	3.21	87.8	35.5	77.6	40.7	38.9	25.7	5.19	0.15	41.3	385	39	20	15	28,5
AD 1	f	AD control	0	0	0	51.5	11.6	74.6	39.8	56.3	21.6	48.4	48.6	66.7	33.3	53.8	13.5	21.8	14.9	1.82	75.3	32.5	79.5	33.9	30	21.6	5.45	0.092	30.9	411	30	77	11	18
AD 1	f	AD DMSO	3	12	2	53.9	12.4	73.7	28.9	66.5	21.4	31.9	61.2	66.7	0	89.4	1,9	32.4	22.7	2.45	83.4	38.6	79.5	40.9	35	24.1	7.73	0.24	62.6	12,019	1432	31	14	18
AD 1	f	AD DMSO	3	11	3	56.5	13.5	75.9	37.6	58.1	18.8	44.3	52.5	100	0	79.2	2,9	15.7	17.7	2.18	82.6	32.5	76.1	37.6	31.5	21.8	6.09	0.14	24.6	10,836	1147	30	17	22
AD 1	f	AD DMSO	2	6	3	54.2	9.52	77.5	40	56	17.6	53.1	43.9	100	0	78.8	9.41	15.3	17.6	2.33	82.8	44.3	80,1	44.9	33.6	23.8	14.4	0.29	23.1	4436	993	23	13	18
AD 1	f	AD DMSO	0	4	0	59.1	24.2	67	35.2	58.8	28.2	40	54	33.3	0	20.6	45.6	12.9	21.2	1.38	83.8	65	85.6	56.3	28.5	19.9	10.3	0.57	46	4350	394	38	18	16
AD 1	f	AD DMSO	0	4	3	51.2	8.8	80.2	35.2	61	15.4	37.6	57.9	0	50	62.7	8.47	17.3	21.6	2.03	88	45.2	82.8	45.4	30	19.3	10.6	0.31	54.5	8621	1520	51	12	22
AD 1	m	AD DMSO	0	10	4	51.4	14.7	72.8	38.7	54.8	22.6	53.3	40.5	33.3	66.7	48.2	24.1	25.7	13.4	1.51	80.6	30.6	80.4	32.2	29.9	20.4	9.97	0.44	22.1	6889	1451	42	24	18
AD 1	f	AD DMSO	0	11	1	61	33.9	56.6	32.2	60.1	37.9	36.3	56	44.4	11.1	78.6	6.35	20.6	17	1.73	83.3	30.7	79.4	34.9	29.6	19.9	12	0.45	15.2	11,580	1728	35	19	17,5
AD 2	f	AD DMSO	5	4	1	59.3	34.5	29.1	43.3	54.6	69.4	36.5	61.8	57.9	15.8	78.9	2.31	21.6	14.5	1.68	69.9	67.2	76.7	51.2	30.5	27	15	0.59	35.9	1884	63	21	21	19,5
AD 2	f	AD DMSO	2	4	1	56,1	32.6	32.4	58.3	40.3	66	32.9	62.6	60	20	54.8	12.2	36.3	20.4	1.42	58.4	59.6	76.7	35.3	20.9	19.7	19	1.84	28.8	1646	49	13	13	16
AD 2	f	AD DMSO	0	4	0	61.7	34.8	28.9	44.4	53.2	69.6	37.2	62.2	66.7	0	92.5	2.01	27.9	13.1	1.81	74.2	68.7	75.4	52.2	35.1	28.8	16.2	0.33	46.8	169	48	12	14	16
AD 2	f	AD DMSO	6	4	0	58.6	27.2	32.7	49.3	48.3	65.7	37.6	61.6	0	0	100	0	25.8	11.2	1.58	75.5	67.6	76	54	34	30.5	13.5	0.53	52.9	349	70	14	15	24
AD 2	f	AD DMSO	4	4	1	59.1	33.4	28.3	42.3	55.4	70.2	39.2	59.7	100	0	86.2	0	19.5	10.4	1.14	74.2	72.2	78.9	54	35.5	29.5	15.4	0.75	50.7	633	55	11	11	13
AD 2	f	AD DMSO	6	4	2	57.5	24.5	30.6	39.7	58.1	68.1	29.8	68.4	0	0	100	0	27.4	12.7	2.86	64	68.2	62.7	45.8	31.8	30.5	9.03	0.43	41.2	456	44	15	15	15
AD 2	m	AD control	0	1	2	65.3	28.6	28	41.8	56.4	70.7	29.3	68.4	0	0	85.7	0	31	16.3	3.38	75.5	72.8	69.4	52.5	38.6	32.8	15.1	0	41.4	NaN	NaN	7	7	9
AD 2	m	AD control	0	0	0	62.1	23.7	30.2	39.4	58.5	68.5	37.2	61.6	0	0	69.2	7.69	32.8	18.4	5.24	78.9	76.5	73.3	65.7	46.7	42.9	17.9	0.83	55.6	2757	39	13	13	14
AD 2	m	AD control	0	1	0	60.3	27.5	31.5	38	60	67.3	31	67.9	0	0	51.9	7.41	25.7	14.1	3.02	66.1	73.9	68	46.2	28.9	29.7	11	2.03	33.3	762	39	9	11	12
AD 2	m	AD control	0	1	0	64.4	29.1	27.5	35.5	62.9	71	34	64.8	0	0	100	0	27.4	13	4.21	67.3	75.1	69.3	52.1	33.6	33.1	18.1	1.13	41.8	10,020	49	12	11	12,5
AD 2	f	AD control	0	1	0	62	24.9	28.1	39	59.1	70.8	30.9	67.5	50	0	100	0	35.2	17.6	3.41	73.4	67.4	64.8	52	29.9	24.3	18.8	1.06	52.6	193	33	13	13	13,5
AD 2	f	AD control	0	2	0	65.7	31	24.4	35.3	62.6	74.4	29.1	68.8	0	0	71.4	0	31.8	17.2	2.61	75	59.9	66.8	51.3	34.6	26.7	17.6	1.62	57.7	5636	32	8	9	10
AD 3	f	AD DMSO	0	4	1	60.8	33	34	51.7	46.2	64.3	33.3	66	0	0	0	100	20,3	20.5	2.61	78.5	60.8	74.2	51.5	27.1	24.5	9.54	1.9	23.2	245	67	13	10	10
AD 3	f	AD DMSO	2	5	0	59.5	36.7	32.6	54.6	42.9	65.6	38.9	60.8	100	0	0	100	19.4	17.4	1.73	80.7	68.6	76.9	55.3	31.7	29.9	11.9	1.44	20.3	55	47	20	11	13
AD 3	f	AD DMSO	0	3	1	60.6	40.1	31.2	53.3	44.6	67.2	36.3	63	0	0	0	0	22	21.9	5.78	82.5	62.5	76.2	57.5	31.9	30.1	13.9	0.79	23.7	997	86	15	12	15
AD 3	f	AD DMSO	0	4	2	59.4	40.3	31.8	54.5	43.1	66.5	40.6	58.7	0	0	0	100	21.3	18.5	2.18	81.3	66.1	75	55.6	33.5	32.8	10.3	1.46	22.5	2462	77	20	16	21
AD 3	f	AD DMSO	0	5	1	53.3	31.9	37	56.2	41.3	61.4	37.7	61.7	0	0	0	100	21.9	20.7	2.39	67.9	42.6	65.3	38.9	19.8	19.9	28.6	2.87	22.5	608	77	15	10	9,5
AD 4	f	AD DMSO	0	3	1	45.6	29.6	35	52.2	46	61,6	27.7	70.3	100	0	33.3	33.3	17.5	15.5	1.03	52.5	60.1	73.3	24.5	9.15	10.9	37.4	5.15	9.49	5089	135	45	15	13,5
AD 4	f	AD DMSO	0	5	0	58.3	21.1	25.6	45.6	52.2	73	33.1	65.8	0	0	0	0	25.1	15.9	1.62	67.3	58.7	77.1	43.4	37.8	35.9	4.03	0.32	31.5	353	158	58	11	13
AD 4	f	AD DMSO	0	6	3	57.8	17.7	27.6	45.1	52.8	70.9	36.5	63.2	0	0	0	0	17.3	13	1.49	67.4	62.6	78.9	44.2	38.6	36.7	8.07	0.15	33.7	1056	109	80	11	14
AD 4	f	AD DMSO	0	6	0	52.3	14.2	31.9	49.4	48.2	66.5	32.6	66.9	0	0	0	0	19.1	14.5	1.59	67.2	64.2	77.8	42.1	28.6	26.8	11.3	2.1	26.4	421	1111	114	13	11
AD 4	m	AD DMSO	0	2	0	51.4	10.7	33.8	53.3	44.5	64.6	31.9	66.5	0	0	0	0	22.6	16.8	1.91	72.5	62.1	78.8	52.7	42.8	37.6	12.1	0.23	52.2	667	180	23	10,5	10
AD 4	m	AD DMSO	0	4	0	55.5	17.9	28.2	50.9	47.4	70.5	26	72.5	100	0	0	0	29.6	20.8	2.43	67.6	58.3	74.7	41.7	38	33.8	9.8	0.82	49	817	99	23	11,5	13
AD 5	f	AD DMSO	1	5	3	84.6	39.9	27.5	22.5	74.1	71.1	39	60.2	100	0	100	0	30.3	16.3	0.98	78.4	21.6	78.1	22.2	48.2	42.4	0.55	0	62.5	209	53	21	26	18
AD 5	f	AD DMSO	2	3	2	88.2	48.4	22.9	17.7	79.2	75.8	38.5	60.8	100	0	58	7.14	29.5	15.1	0.93	79	21.9	76	20.5	47.2	39.7	0.54	0	62.4	140	60	19	25	17
AD 5	f	AD DMSO	0	5	1	88.5	53.9	21.6	15	80.8	77.1	37.5	61	72,7	0	49.9	10.9	47.6	31.2	0.81	78.2	19.1	69.6	19.9	37.6	31.7	0.39	0	62.9	1816	361	23	26	18
AD 5	f	AD DMSO	0	6	1	88.7	53.5	22.6	15.4	79.1	75.8	29.3	68.3	95,5	0	50.7	14	52.3	31.6	1,01	84.5	30.3	79.5	26.6	41.4	33.1	0.78	0	75.1	560	198	20	20	14
AD 5	f	AD DMSO	0	7	2	86.4	50.2	23.4	19.3	73.8	74.9	27.4	66	100	0	36	28.1	30.5	33.6	1.33	85.1	36.5	79	32	41.3	33.4	1.18	0	77.3	222	234	20	22	16
AD 5	f	AD DMSO	0	8	1	87.7	48.5	23	18	78.8	75.8	27.7	70.4	100	0	49.8	15.9	46	24.2	1.06	78.7	40.8	77.7	30.6	65.9	58.1	1.86	0	70.3	179	299	27	26	18
AD 6	f	AD DMSO	4	6	0	81.2	53.7	37.9	18.2	75.2	57.6	31.5	61.2	71.9	5.26	75	0	26.6	17	1.93	80.6	63.4	87.9	46.7	24.8	22.2	37.8	0.45	49.7	737	81	12	11	9
AD 6	f	AD DMSO	0	5	0	81	55.8	34.1	15.1	78.3	61.6	17.2	79	100	0	84.2	0	39.1	35	1.43	82.6	57.1	90.3	48.1	26.5	24.1	48.5	0.51	46.6	1118	159	13	11	10
AD 6	f	AD DMSO	2	4	0	80.2	50.2	41.8	19.1	75.5	53.5	38.1	55.6	69.4	11.1	66.7	33.3	16.7	16	1.63	80.2	59.3	87.8	45.2	19.6	21.3	40.2	0.56	49.7	745	49	24	14	11
AD 6	f	AD DMSO	8	6	0	82.6	60.7	32.5	15.6	80.2	64	39.4	56	69.8	2.33	71.4	0	11.1	16	1.57	79.1	64.7	86.9	46.9	23.3	21.1	34.5	0.4	51.3	591	100	19	13	11
AD 6	f	AD DMSO	0	5	0	81.8	52.5	41.9	22.5	69.3	51.7	25	64.2	68.4	7.89	90	0	13.5	26	1.16	84.5	49.2	92	39.4	21	19.9	48.6	0.51	72.8	1108	104	25	22	16
AD 6	f	AD DMSO	0	3	0	77.1	44.9	41.9	22.6	72.6	51.8	45.7	48.2	77.3	2.67	34.6	15.4	23.2	11.6	1.33	77.3	63.3	87.9	43.2	18.6	17.8	32.7	0.4	34	1297	1039	16	11	9
AD 7	f	AD DMSO	6	6	1	67.6	73.7	19.7	45.2	51	78.9	23.1	74.8	100	0	89	3.6	14.6	46.3	9.09	65.4	43.7	43	37.7	29.1	37.1	43.6	0.52	13	2582	253	15	15	9,5
AD 7	f	AD DMSO	4	7	1	70.6	74.3	19	40	55.5	79.4	21.8	75.4	33.3	0	93.6	2.95	19.9	48.7	10.8	63.7	36.7	40	32.5	25.1	34.5	42	0.43	15.9	1521	144	14	14	12
AD 7	f	AD DMSO	0	5	1	71.7	74.4	17.5	46.6	51.5	81.8	21.5	77.6	0	0	96.3	0.83	22	50.8	13.8	79.3	60.9	44.9	54.5	32.2	40.2	45.4	0.11	15.6	1484	174	17	16	20
AD 7	m	AD DMSO	0	5	0	70.8	73.5	19.1	44.5	51.3	79.4	22.5	76.1	0	0	99.8	0	26.1	48.8	12.1	68.9	46	43.7	40.7	27.7	37.6	43.7	0.092	17.2	1098	917	17	17	13
PV 1	m	PV control	0	0	0	65.4	17.9	76.2	35	56.6	18.8	34.5	52.7	50	0	25.9	41.4	40.9	24.6	2.97	81.6	50,1	46	45.3	31	37.3	18,1	2.59	42.1	171	27	38	11	24
PV 1	m	PV control	0	0	0	67.9	29.3	70.5	34.3	57,2	22	41.6	44.9	20	20	24.8	28.6	48.9	25.1	2.5	82	64,5	30,8	51.1	27.1	25.3	30.4	2.76	47.7	405	40	46	16	41
PV 1	m	PV control	0	0	0	67.9	24.9	75	31.4	57.2	19.7	32.4	54.9	40	0	27	39.7	46.7	28	2.37	76.2	50,5	30,1	40.5	27.5	29.7	24.2	3.13	38.3	67	26	199	16	108
PV 1	m	PV control	1	0	0	68.8	30.4	73	29.4	62.1	21.6	35.1	53.9	0	0	35.7	33.7	49.9	21.7	1.58	74.4	45,8	28,6	38.7	19.5	26.6	30.3	2.85	49.7	84	30	123	16	62
PV 1	m	PV control	0	1	0	63.9	18.6	79.8	41.1	52	15.7	51.2	37.8	25	12.5	35.3	17.6	32.2	18.8	1.9	77.9	46,8	42,9	39,9	25	31.8	20.9	2.43	42.6	145	28	82	15	37
PV 1	m	PV control	0	0	0	50.3	30.6	63.9	49.8	42.2	32.1	41.5	38.5	9.09	36.4	13.8	41.5	30.7	20.1	1.57	84	73	19.9	56.5	9.27	60.8	57.4	16.6	19.6	586	45	57	13	32
PV 1	f	PV DMSO	4	8	1	66.2	26.8	74.7	47.4	47	19.3	43.5	41.8	10.4	19.8	18.5	37.4	42.8	16.9	1.85	82	60.3	32.9	50.7	25.4	40.2	31.8	6.28	43.3	6242	69	33	15	76
PV 1	f	PV DMSO	0	3	0	52.5	26.5	62	49.4	45.2	28.2	37.8	38.1	20.9	19.4	14	44.4	75.4	23.6	1.22	78.4	64.5	21.1	48	14.4	62.1	44.5	12.4	31.1	743	162	50	14	41
PV 1	f	PV DMSO	6	3	0	67.8	18.2	78.6	38.1	55.5	16	46.5	45.4	25	25	100	0	74.3	22	1.66	80.2	58.7	42.7	50.5	36.2	22	11.7	1.84	70.8	560	198	55	23	49
PV 1	f	PV DMSO	4	7	0	68.3	19.2	77.5	37.7	55	16.6	44.9	46	26.7	26.7	66.7	0	65.2	20.7	1.84	83.3	56.7	59.4	54.6	35.9	28.1	28.7	1.87	68.5	232	336	49	15	37
PV 1	f	PV DMSO	0	2	0	71	31	74.1	37	54.8	20.2	46.4	43.3	16.7	25	13.5	45.2	42.6	24.4	1.55	80.7	39.1	52.4	40.4	29.9	21	15.4	4.21	41.6	436	70	38	14	28
PV 1	f	PV DMSO	0	2	0	66.4	28.2	73.1	37.9	54.5	21	38	47.5	25	62.5	17.4	36	71.8	21.3	2.06	84.3	57.2	38.6	50.2	25.9	31.9	46.1	8.95	47.3	144	209	79	23	67
PV 2	f	PV control	0	0	0	64.2	13.4	78.2	45.2	50.2	18	60	36	0	0	0	0	10.8	8.51	1.42	66.9	23.9	53	17.6	25.5	0.04	4.94	0	18.3	44	23	38	11	15
PV 2	f	PV control	0	0	0	69.7	25.6	68.6	32.8	57.9	25.2	29.5	58.8	0	0	0	0	13	17.9	1.96	70.1	16.4	49.8	15.2	25.3	0.13	8.48	0.57	18.6	86	49	144	42	127
PV 2	f	PV control	1	0	0	69	22.2	72.2	33.5	60.2	22.4	42.4	50.3	0	0	0	0	14.9	18.1	2.31	74.7	30.6	54.5	27	32.7	0	3.16	0.066	31	230	31	56	15	29
PV 2	f	PV control	0	2	0	67.6	16.9	75	38.5	56.9	20.6	53.9	42.6	0	0	0	0	11.4	12.6	1.66	69.5	31.1	53	23.7	24.5	0.038	6.67	0.056	22.5	716	31	30	15,5	23
PV 2	m	PV control	0	0	0	67	16	75.3	39	55.6	20.7	53.5	40.1	0	0	0	0	11.6	9.7	1.56	66.1	28.4	50.2	21.6	26	0.029	3.79	0.21	22.9	148	31	30	13	29
PV 2	m	PV control	0	0	0	65.9	17.4	76	42.7	52.4	20.8	63.3	31.7	0	0	0	0	9.08	8.78	1.19	67.2	28.6	53.7	22.4	23.6	0.072	4.59	0.25	56.2	58	21	55	15	25
PV 2	m	PV DMSO	0	6	1	66.5	19.8	67	33.9	58	27.9	25.4	60.9	0	0	0	0	67.9	24	2.63	77.3	29.1	41.8	24.4	30.6	0.018	9.05	0.16	74.7	305	3093	37	11	24
PV 2	m	PV DMSO	0	4	1	64.4	13.7	77.5	44.1	51.2	18.8	62.5	32.5	0	0	0	0	49.4	10.2	1.6	72.5	33.2	52.9	27.3	27.6	0	5.23	0.13	64.9	833	1432	105	16,5	33
PV 2	m	PV DMSO	4	8	3	66.6	14.3	78.9	42.7	53	17.1	52.4	43.4	0	0	0	0	51.3	10.8	1.94	74.8	25.1	57.5	23	31.3	0.035	5.5	0.071	67.5	157	188	25	12	24
PV 2	m	PV DMSO	6	3	4	61.5	27.1	63.7	35	59.9	32.1	55.9	36.5	0	9.09	0	0	22.2	19.9	1.45	74	28	47.8	24.4	22.3	0.051	13.6	0.33	43.4	317	45	62	14	27
PV 2	m	PV DMSO	0	2	1	69.3	20.9	71.8	36.2	58,7	21.9	52	41.8	0	0	0	0	50,6	11,8	3,56	27,3	76,2	36	6,99	32,1	0,034	8,44	0.15	66.1	874	906	133	16	33
PV 2	m	PV DMSO	0	2	0	67.5	21.8	72.8	36.5	58.5	21.1	48.1	45.8	0	0	0	0	57,1	16,3	3,59	76,6	31	58	29,3	34,2	0	9,52	0.12	66.6	176	255	33	15	26
PV 3	f	PV DMSO	0	4	0	66.2	31.5	66.9	33.3	61.2	27.9	42.3	51.9	0	50	0	100	54.8	28.1	4.18	76.9	9.49	48.5	8.7	27.8	0.048	7.6	1.4	60.5	992	161	19	12	25
PV 3	f	PV DMSO	0	4	1	67.9	34.2	60.6	29.7	64.5	33.5	40.9	52	0	14.3	0	0	54	27.9	6.22	30.6	52.8	50.8	8.29	34.5	0	8.58	1.1	69	706	54	37	12,5	27
PV 3	f	PV DMSO	0	4	2	66.2	31.3	63.7	37	58.4	31.5	59.3	36.8	0	14.3	0	0	48.1	20	4.11	76.2	7.9	50.4	5.89	28.9	0.15	5.46	0.53	65	469	352	53	25	52
PV 3	m	PV DMSO	0	8	3	68.7	33.1	62.8	33.2	61.9	32.6	41.5	52.4	0	0	100	0	56.1	28.1	3.72	78.5	6.25	52.6	6.4	24.8	0	9.01	1.32	68.3	590	455	57	22,5	37
PV 3	m	PV DMSO	0	7	1	64.8	33.9	62.3	29.9	64.4	32.2	45.8	47.5	0	37.5	0	0	56.5	27.1	4.37	80.3	9.54	52.8	8.85	32.4	0.32	8.78	0.92	67.5	1417	124	28	11	22
PV 3	m	PV DMSO	0	4	1	61.8	28.4	63.4	36.6	58.9	32	63	32.5	0	0	0	50	48.4	16.8	3.24	79.5	8.42	57.5	6.41	34.1	0.16	6.39	1.22	71.2	2207	1394	31	26,5	51
PV 4	f	PV DMSO	2	4	3	47.7	24.4	22.6	35.9	62.2	76.3	43.5	55.5	0	0	0	0	18,6	10,2	2,04	49,4	76,2	23,7	31	11,9	17,3	10,3	0.41	26	1203	1149	21	11	18
PV 4	f	PV DMSO	0	6	1	44.3	22.8	24.7	42.2	55.4	74.2	44.8	52.9	0	0	100	0	38.3	16.2	2.87	70.8	74.6	33.4	50.5	23.8	28.2	22,2	2.11	58.7	1338	1511	21	11	15
PV 4	f	PV DMSO	0	6	2	47.1	21.5	26.5	35	62.4	72	42.8	55.6	75	12.5	0	0	40	15.3	2.42	72.9	79.2	43.6	51.9	20.6	26.5	22.4	2.51	62.9	3129	1448	17	10	15
PV 4	f	PV DMSO	0	7	1	41.3	14.9	29.5	39.1	58.5	68.9	55.6	43.4	85.7	0	100	0	25.9	11.2	1.41	63.6	71.1	32.8	42.7	20.3	23.2	14.4	1.92	49.4	287	23	19	21	29
PV 4	f	PV DMSO	4	5	1	39.1	8.72	32.4	39.3	58.6	65.9	48.4	50.6	100	0	0	0	17.8	9.67	1.85	52.5	68.8	20.5	33.5	21.3	23.3	14.9	1.88	49.5	1343	656	29	16	29
PV 4	f	PV DMSO	6	6	1	38.6	16.2	29	37.9	60	69.4	48.5	49.6	100	0	100	0	44.6	18.1	2.11	61	72.5	27.7	40.6	24.5	25.2	15.1	1.57	67.9	1639	1086	20	19	35
H	f	H DMSO	0	3	0	64.6	40.3	61.3	26.7	62.7	33.9	23.7	55.4	37.5	0.78	34.4	19.9	58.4	33.5	1.36	73.3	36.8	69.9	30.1	26.8	1.98	9.29	0.58	8.65	594.5	104	11	11	12
H	f	H DMSO	0	2	0	62.7	38.1	64.5	24.8	65.5	29.3	24.1	58.8	21.2	6.06	45.9	17	49.5	15.6	1.41	56.7	31.2	72	18.5	26.5	1.96	14.8	0.87	15.6	239.5	101	9	8	7,5
H	f	H DMSO	0	2	0	62.2	41.5	64.6	28.8	63.3	30.8	33.1	54.4	34	5.66	49.2	17.5	36.1	9.6	1.51	47.1	26.5	71.5	12.3	29.1	2.35	18.6	0.95	16.7	1903	59	7	7	8,5
H	f	H DMSO	0	3	0	57.2	42.5	51.9	27	60.9	41.9	12.4	60.8	29	7.53	48.8	18.4	3.19	9.62	2.19	37.6	17.4	60.3	8.01	15.3	3.26	28.7	3.98	6.62	556	76	9	8	9
H	f	H DMSO	0	4	0	63.7	51.8	47.9	20.2	65	45.8	8.46	69.1	12.4	2.06	56	16.5	7.51	18.7	1.09	60.6	26.3	57.8	17.8	17.6	2.91	31.4	3.52	8.68	597	53	7	7	8
H	f	H DMSO	0	6	0	62.9	36.5	64.2	29.3	64	31.2	34.4	52	18.3	1.22	20	30	3.66	3.97	1.25	52.3	31.7	65.9	15.9	21.1	2.44	19.9	3.22	9.79	191	54	8	8	8
H	f	H control	0	2	0	64	36	63	28.3	62.7	30.8	35.5	48.7	13.7	1.37	12	50	43	24.1	1.15	55.8	36.5	65.1	23	29.1	1.81	13.8	0.66	13.7	376	31	11	9	11
H	f	H control	0	2	0	68.1	36.3	57.9	28.6	62.4	37.2	45.9	36.5	38.9	4.44	17.7	39.6	14.5	14.1	1.36	69	40.8	70.3	33	26.3	1.96	15.9	1.26	31.5	74	38	13	10	12
H	f	H control	0	2	0	63.7	42.5	59.8	28.3	60.1	35.1	29.7	44.2	23	2.03	15.4	37.9	32.6	15.3	1.33	65.9	36.3	64.1	25.3	20.5	2.64	19.1	1.82	41.9	143	32	13	12	16
H	f	H control	0	2	0	64.5	45.5	59.9	26.6	62.9	34.5	31.3	49.2	24.5	1.89	16.7	39.2	9.96	17.3	1.58	62.2	39.8	64.2	29.1	26.6	1.85	22.4	1.55	18.5	8	10	13	8	9,5
H	f	H control	0	2	0	55.6	33.4	64	27.6	64.2	30.8	32.6	50.6	25	1.67	12.2	44.6	8.39	12	1.1	62.9	34.3	64.2	25.8	22.6	1.31	18	1.75	72.5	18	11	12	9	9
H	f	H control	0	2	0	64.7	40.1	66.1	26.5	66.1	29	37.7	51.1	16.7	16.7	9.42	24.6	8.9	7.2	1.28	68.8	38.7	71	30.2	27.1	1.56	12.7	0.69	39.8	7	10	8	8	10
Nonreconstituted	f	Non reconstituted	0	1	0	NaN	NaN	NaN	NaN	NaN	NaN	NaN	NaN	NaN	NaN	NaN	NaN	NaN	NaN	NaN	NaN	NaN	NaN	NaN	NaN	NaN	NaN	NaN	NaN	336	226	36	11	10
Nonreconstituted	f	Non reconstituted	2	1	2	NaN	NaN	NaN	NaN	NaN	NaN	NaN	NaN	NaN	NaN	NaN	NaN	NaN	NaN	NaN	NaN	NaN	NaN	NaN	NaN	NaN	NaN	NaN	NaN	120	106	36	12	14
Nonreconstituted	f	Non reconstituted	0	1	0	NaN	NaN	NaN	NaN	NaN	NaN	NaN	NaN	NaN	NaN	NaN	NaN	NaN	NaN	NaN	NaN	NaN	NaN	NaN	NaN	NaN	NaN	NaN	NaN	461	77	66.5	13	16
Nonreconstituted	f	Non reconstituted	0	0	0	NaN	NaN	NaN	NaN	NaN	NaN	NaN	NaN	NaN	NaN	NaN	NaN	NaN	NaN	NaN	NaN	NaN	NaN	NaN	NaN	NaN	NaN	NaN	NaN	174	128	66	12	15

Abbreviations: AD, atopic dermatitis; f, female; H, healthy; ISD, inflammatory skin disease; m, male; MCP3, monocyte chemoattractant protein 3; NaN, not a number; PV, psoriasis vulgaris; Treg, regulatory T cell; TSLPR, TSLP receptor.

**Figure 6:**
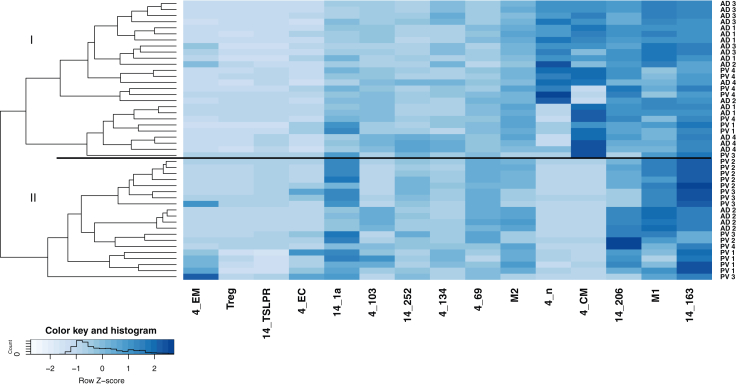
**Illustration of the comparison of immune profiles between NSG-AD and NSG-PV mice.** The mice were treated as described in the previous section, and frequencies of human immune cells isolated from mouse spleens were analyzed by flow cytometric analysis (NSG-AD: n = 4, n = 22; NSG-PV: n = 4, n = 24). AD, atopic dermatitis; PV, psoriasis vulgaris; Treg, regulatory T cell; TSLPR, TSLP receptor.

**Figure 7:**
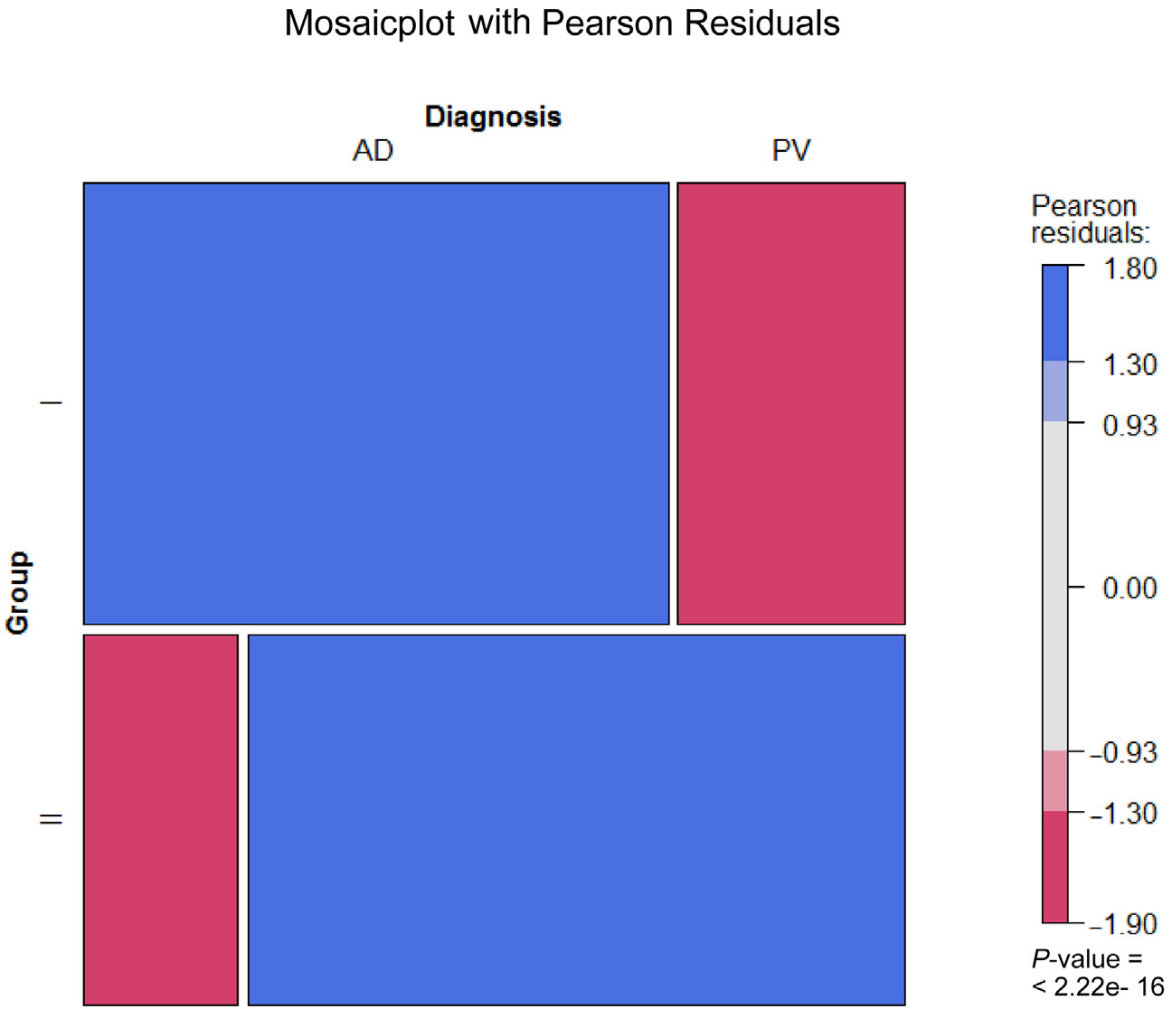
**Mosaic plot and Pearson residual contingency analysis of the immunological profiles in mice.** The width of the rectangle indicates the number of samples gained from mice reconstituted from patients with AD or PV. Red tiles indicate significant negative residuals, where the frequency is less than expected. Blue tiles indicate significant positive results, where the frequency is greater than expected. Labels on the right side indicate the contribution of each cellular profile to the significance of the chi-squared test result. AD, atopic dermatitis; PV, psoriasis vulgaris.

**Figure 8:**
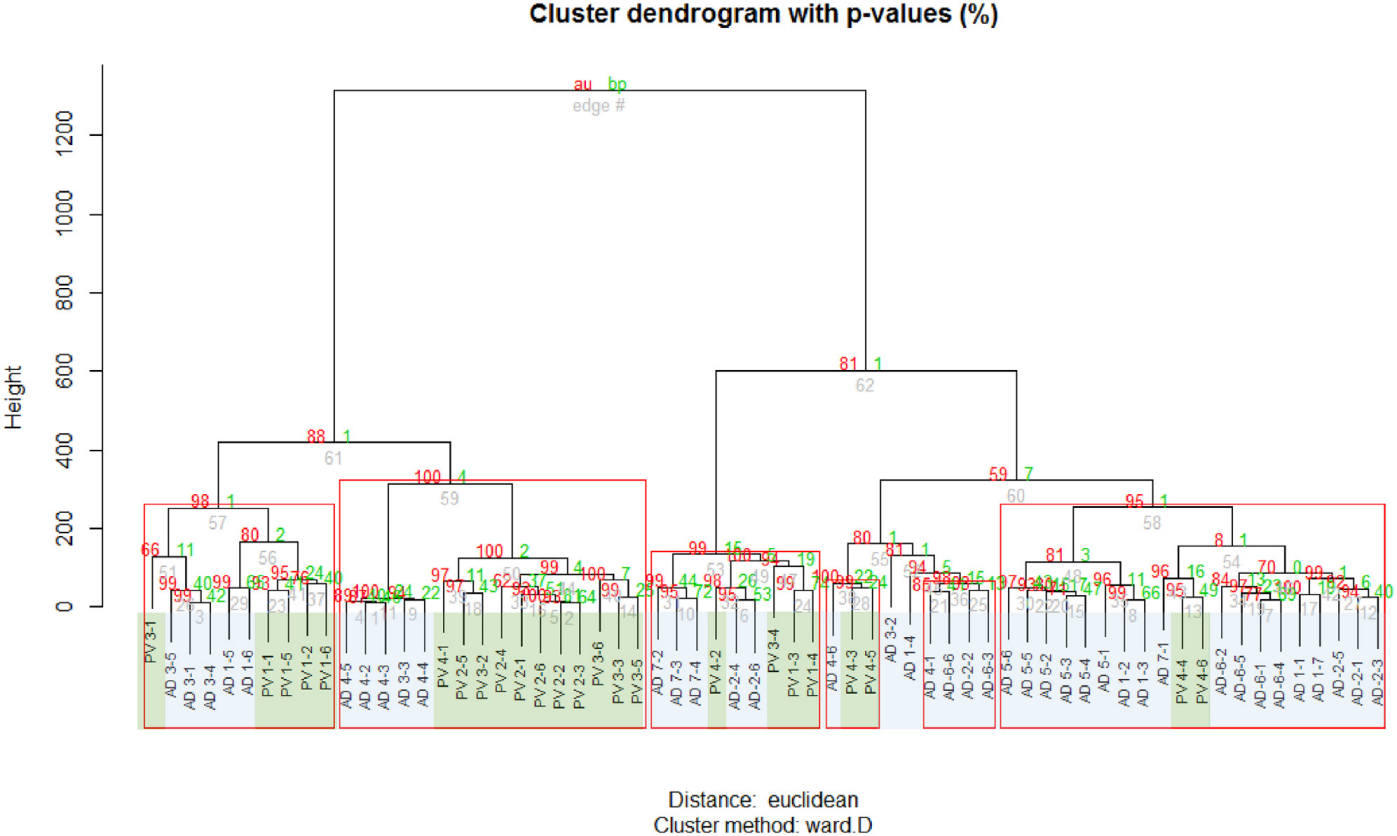
**pvclust illustration of the comparison of immune profiles between NSG-AD and NSG-PV mice.** The mice were treated as described in the previous section, and frequencies of human leukocytes from mouse spleens were analyzed by flow cytometric analysis (NSG-AD: n = 4, n = 22; NSG-PV: n = 4, n = 24); red rectangles show branches with a *P* < .05. AD, atopic dermatitis; PV, psoriasis vulgaris.

To assess the extent to which the immunological profile could be associated with the diagnosis of the respective donors in the NSG mice, a hierarchical cluster analysis was conducted using the frequencies of human leucocytes isolated from mouse spleens. As depicted in Figure 6, 18 of 22 NSG-AD mice clustered in group I, whereas 17 of 24 NSG-PV mice clustered in group II. In addition, mice from the respective studies clustered closely together, indicating that the specific immunological background was partially preserved. The mosaic plot confirmed the statistical analysis (Figure 7). To further support this observation, another cluster analysis was performed using pvclust (Figure 8).

Furthermore, all 4 AD donors selected for reconstitution, whose immunological profiles were examined and found to cluster in the AD group, gave rise to NSG-AD that also clustered in the AD group. However, there was one exception: the PV donor, whose immunological profile clustered with other PV donors, resulted in NSG-PV mice that clustered with other NSG-AD mice (Figure 1b).

**Figure 9:**
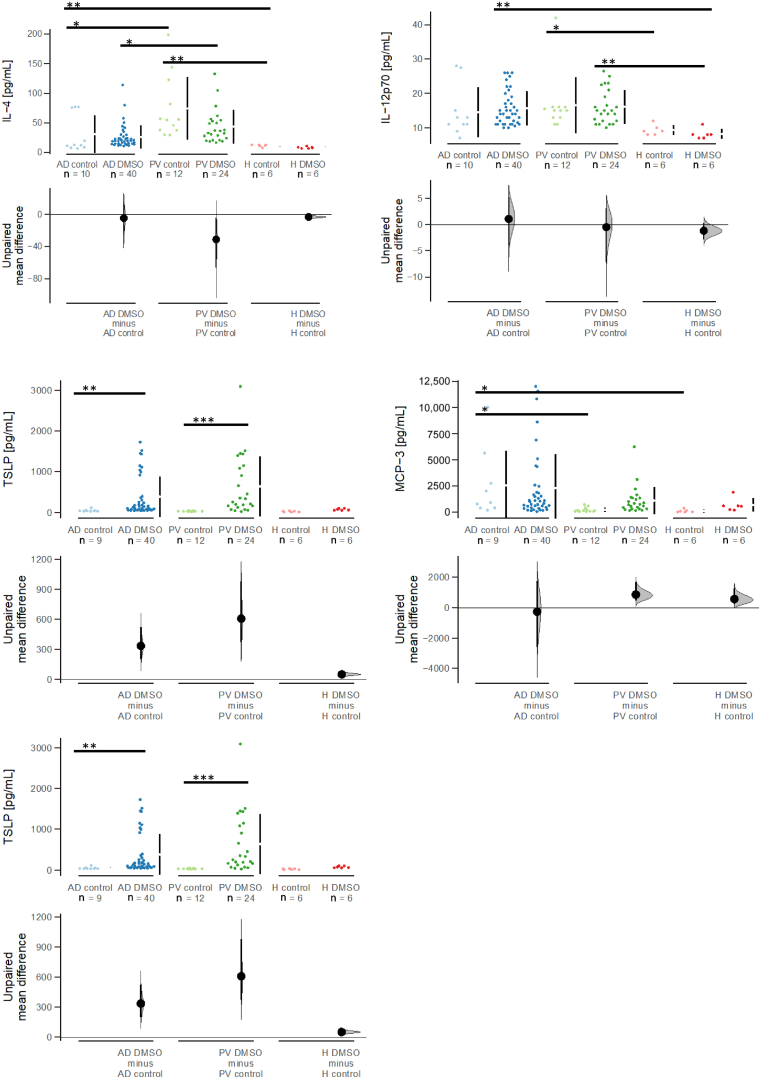
**Display of the expression of human cytokines and mouse MCP3, which differ between the skin of NSG-AD and NSG-PV mice.** The mice were treated as described in the previous section, and proteins were extracted from the skin for analysis using Luminex assays. The cytokines and MCP3 are presented as Cumming plots. ∗*P* = .05– .01, ∗∗*P* = .01– .001, and ∗∗∗*P* < .001. AD, atopic dermatitis; H, healthy; MCP3, monocyte chemoattractant protein 3; PV, psoriasis vulgaris.

To investigate whether the differences between the 2 models were also reflected in the expression of cytokines, proteins were extracted from the skin and analyzed by Luminex analysis (Figure 9). No significant difference was observed in all examined cytokines between the respective control and DMSO-sensitized groups, and human IL-12 appeared to play a role in mediating inflammation, but levels were not significantly different among the groups. However, a significant difference was detected when levels of human IL-4 and human IL-17 were examined. Human IL-4 levels were significantly higher in the PV DMSO group than in the AD DMSO group (AD DMSO vs PV DMSO: *P* = .002, Wilcoxon rank sum test with continuity correction). On the other hand, human IL17A levels were higher in the PV control and the PV DMSO groups than in the AD control and AD DMSO groups (AD control vs PV control: *P* = .003; AD DMSO vs PV DMSO: *P* < .001, ANOVA). Monocyte chemoattractant protein (MCP3), previously identified as an inflammatory marker in the NSG-ulcerative colitis mouse model (Unterweger et al, 2021b), was significantly induced in the NSG-PV mouse model upon sensitization with DMSO (PV control vs PV DMSO: *P* = .02, ANOVA). Although levels of MCP3 were higher in the AD DMSO group, the difference was not significant. In all analyses, NSG-H mice exhibited low levels of cytokines, corroborating the analysis of the scores.

## Discussion

In this study, we demonstrated significant differences in the inflammatory profiles and immune cell networks between patients with AD and those with PV. These differences were consistent with previous findings of high heterogeneity among patients with AD and a predominant Th2/Th1 cell–driven response in AD (Brunner et al, 2017). In contrast, the PV group exhibited elevated frequencies of macrophages, activated macrophages, activated monocytes, and M1/M2 monocytes, suggesting a monocyte- and macrophage-driven inflammation in these patients.

The network analysis further supported these differences, showing distinct equilibria between AD and PV. The AD network displayed higher heterogeneity with Th17 and Th1/Th17 cells at the center, whereas Th1 and Th2 cells were located at the periphery. These observations are in line with those of previous studies that suggest a role of Th2/Th1 and Th17 cells depending on the status and subgroup of the disease (Bieber, 2022; Suárez-Fariñas et al, 2013). A significant cell hub consisted of CD14+ TSLP receptor–positive cells, which have been identified as a therapeutic target in AD (Nakajima et al, 2020). The location of CD1a-expressing monocytes and macrophages also differed between the 2 networks, with the AD network embedding these cells and the PV network placing CD1a-expressing monocytes at the outskirts. In the epidermis, these cells referred to as inflammatory dendritic epidermal cells are known to express increased frequencies of the high-affinity IgE receptor and the mannose receptor (CD206) in addition to CD1a. Owing to these characteristics, inflammatory dendritic epidermal cells are considered important mediators in AD and atopic eczema (Wollenberg et al, 2002, 1996). Furthermore, inflammatory dendritic epidermal cells have been utilized to discriminate between intrinsic and extrinsic AD (Oppel et al, 2000).

In contrast to the AD network, the PV network was densely centered on activated monocytes and macrophages, with TSLP receptor–expressing monocytes located at the periphery, indicating minimal correlation with other cells. Th1/Th17 cells but not Th17 cells alone were embedded in the network, suggesting that in PV, Th1 cells derive from Th17 cells. This plasticity has been shown to be crucial in the pathogenesis of colitis (Harbour et al, 2015).

Reconstitution of NSG mice with PBMCs from donors with AD or PV, followed by challenge with DMSO, resulted in distinct pathological manifestations and expression of inflammatory markers. NSG-AD mice exhibited a more inflammatory phenotype, partially reflecting the characteristics of the immunological profile of patients with AD. The predominant features of the NSG-AD model were the influx of T and B cells as well as monocytes into the dermis and frequently into the epidermis, along with elevated levels of MCP3. In contrast, the influx of lymphocytes in NSG-PV mice was less pronounced than in NSG-AD mice, but increased presence of fibroblasts and higher levels of IL-17A were observed.

In both models, the application of DMSO was necessary for the development of symptoms and skin pathologies. The exact mechanism through which DMSO triggers the reaction is unclear. DMSO is known to penetrate the skin and act as a carrier (Brayton, 1986), which could potentially enable skin-residing bacteria or fungi to induce the inflammatory response. However, DMSO is also recognized as a skin irritant that can cause burning sensations, erythema, and itching. Regardless of the mechanism through which DMSO triggers the response, the development of symptoms and pathological manifestations requires the immunological background of a diseased donor. The same observation has previously been made in other models (Weß et al, 2023).

The analysis of splenic leucocytes provided insights into the impact of reconstituted PBMCs in the NSG mice. Regardless of the challenge, a significant difference was observed between the NSG-AD and NSG-PV groups. Similarly, levels of ILs such as IL-4, IL-12, and IL-17A in the skin did not increase upon challenge but exhibited differences between NSG-AD and NSG-PV mice. No increase in cytokines was observed in the NSG-H mice, corroborating the previous observations.

Hierarchical cluster analysis demonstrated that the immunological profiles of most mice clustered according to the diagnosis of the donor, with most mice from the same study closely clustering together. This finding supports previous studies that have suggested a partial conservation of the immunological profile of the donor in the mice (Jodeleit et al, 2020; Unterweger et al, 2021b).

Therefore, the NSG-AD and NSG-PV models help bridge the gap to human diseases and are well-suited for elucidating immunological processes and validating novel therapeutics.

### Limitations of the study

This study has certain limitations, including a small number of patients, which provides only a snapshot of these patient populations. The study also does not account for the dynamic nature of inflammation and may not be suitable for defining patient subgroups. Furthermore, the selected subtypes of immune cells do not cover the entire spectrum of cells, potentially missing important factors. Despite these limitations, the study provides valuable insights and suggests that this approach can contribute to a better understanding of the underlying immunological processes.

The limitations of the animal model are evident as well. Although the chimeric NSG mice used in this study are more representative of human diseases than conventional models, they still require the interaction of mouse chemokines with human leucocytes. The compatibility of chimeric ligands and their respective receptors is not yet fully understood. In addition, the reconstituted PBMCs lack polynuclear leucocytes, which may play a significant role, particularly in AD.

In summary, the combination of profiling and preclinical studies in NSG-AD and NSG-PV mice has the potential to enhance our understanding of the immunological processes underlying the diseases. Furthermore, it allows for the evaluation of therapeutics targeting specific molecular targets in humans.

## Materials and Methods

### Isolation of PBMC

A total of 20–60 ml of peripheral blood in trisodium citrate solution (S-Monovette, Sarstedt) was collected from the arm vein of patients with AD and PV following a previously described protocol (Jodeleit et al, 2020).

The collected blood was diluted with Hank’s balanced salt solution (Sigma-Aldrich) in a 1:2 ratio and loaded onto LeucoSep tubes (Greiner Bio-One). The tubes were then centrifuged at 400*g* for 30 minutes without acceleration and break. PBMCs were extracted from the interphase and diluted with PBS to a final volume of 40 ml.

The cell suspension was then counted and centrifuged at 1400*g* for 5 minutes. The resulting cell pellet was resuspended in PBS at a concentration of 4 × 10^6^ cells in 100 μl, ready for further experimentation.

### Flow cytometry analysis

All antibodies listed in Table 6 were acquired from BioLegend and utilized following the manufacturer’s instructions. Flow cytometry analysis was conducted using a Thermofisher Attune NxT instrument from Thermo Fisher Scientific. The resulting data were analyzed using FlowJo 10.1 Software developed by FlowJo LLC. For gating strategy see Supplementary Figure S1.

**Table 6:** List of Antibodies Used to Label Human Leukocytes

Surface Marker	Color	Cat #, RRID #
Flow cytometric analysis		
CD4	APC- Cy7	BioLegend, Cat# 317417, RRID:AB_571946
CD45RA	PE/Cy7	BioLegend, Cat# 304126, RRID:AB_10708879
CD45RO	PE	BioLegend, Cat# 304206, RRID:AB_314422
CD62L	FITC	BioLegend, Cat# 304838, RRID:AB_2564162
CCR7	APC	BioLegend, Cat# 353214, RRID:AB_10917387
CXCR3	FITC	BioLegend, Cat# 353704, RRID:AB_10983066
CCR6	PE/Cy7	BioLegend, Cat# 353405, RRID:AB_10918985
CCR10	APC	BioLegend, Cat# 341505, RRID:AB_2291025
CCR4	PE	BioLegend, Cat# 359411, RRID:AB_2562432
CD8	PerCP-Cy^TM^ 5.5	BioLegend, Cat# 344750, RRID:AB_2687201
CD103	APC	BioLegend, Cat# 350215, RRID:AB_2563906
CD14	APC-Cy7	BioLegend, Cat# 325619, RRID:AB_830692
CD64	PerCP-Cy^TM^ 5.5	BioLegend, Cat# 305023, RRID:AB_2561585
CD163	FITC	BioLegend, Cat# 333617, RRID:AB_2563093
CD206	APC	BioLegend, Cat# 321109, RRID:AB_571884
CD252	Biotin	BioLegend, Cat# 326306, RRID:AB_2303694
TSLPR	APC	BioLegend, Cat# 322807, RRID:AB_2085327
CD1a	Biotin	BioLegend, Cat# 300112, RRID:AB_389344
CD80/86	PE/Dazzle	BioLegend, Cat# 305229, RRID:AB_2566488
CD69	FITC	BioLegend, Cat# 310903, RRID:AB_314838
CD25	PE/Cy7	BioLegend, Cat# 302611, RRID:AB_314281
CD134 (Ox40)	PE	BioLegend, Cat# 350003, RRID:AB_10641708
IgD	APC/Cyanine7	BioLegend, Cat# 348217, RRID:AB_11204072
CD27	PE	BioLegend, Cat# 124209, RRID:AB_1236464
CD38	PE	BioLegend, Cat# 356603, RRID:AB_2561899
IHC		
Anti-hu CD4 RPA-T4		Thermo Fisher Scientific Cat#14-0049-82, RRID:AB_467077
Anti-hu CD8		Thermo Fisher Scientific Cat# 14-0008-82, RRID:AB_2572848
Anti-hu CD14		Thermo Fisher Scientific Cat# 14-0149-82, RRID:AB_467129
Anti-hu CD19		Thermo Fisher Scientific Cat# 14-0190-82, RRID:AB_11219274
Mouse IgG1 Isotype control		Thermo Fisher Scientific, Cat# 14-4714-82, RRID:AB 470111
Rabbit anti mouse	Alexa Fluor 488	Thermo Fisher Scientific Cat# A-11059, RRID:AB_2534106

Abbreviations: #, number; APC, allophycocyanine; Cat, catalog; hu, human; IHC, immunohistochemistry; PE, phycoerythrin; RRID, Research Resource Identifier.

### Study protocol

Mice used in this study were sourced from Charles River Laboratories and housed under specific pathogen-free conditions in individually ventilated cages. The facility adhered to the guidelines set forth by the Federation of Laboratory Animal Science Association.

The experimental mice were NSG (NOD.cg-Prkdc^SCID^Il2rg^tm1Wjl^/Szj) mice, aged 6–8 weeks. On day 1, the mice were engrafted with a 100 μl cell solution through the tail vein, following a previously described procedure (Jodeleit et al, 2020, 2018). The mice were assigned to either the unchallenged control group or the experimental group, where they were shaved and depilated with Veet depilatory cream on both sides (2 × 1 cm) under isoflurane anesthesia on days 8, 16, and 20. In addition, they were challenged with 100% DMSO (Sigma-Aldrich) on days 8, 16, 18, and 20. The mice were killed on day 21 for further analysis.

### Clinical inflammatory skin disease score

The severity of clinical symptoms and skin symptoms was assessed on days 8, 16, 18, and 20 using the following criteria: 0% (0 points), 0–5% (1 point), 5–10% (2 points), 10–15% (3 points), and 15–25% (4 points) for loss of body weight; normal (0 points), reduced activity (1 point), somnolence or shaking (3 points), and apathy (4 points) for behavior; ruffled fur for up to 2 days (1 point), ruffled fur for >2 days (2 points), and barbering (2 points) for fur; hunched posture (4 points) for body posture; redness or dandruff or wet (2 points) and eczema or bloody crusting (4 points) for skin; and mild (1 point), medium (2 points), and severe with sleeping disorders (4 points) for itching.

The scores for each category were added together to obtain a total score, with a maximum of 22 points per day. Mice with a severity score of ≥4 were immediately killed and not included in the analysis. All scores were collected for statistical analysis.

### Macroscopical inflammatory skin disease score

On the day of necropsy, a photograph of the shaved and depilated skin was taken, and the following criteria were used for scoring: redness (1 point), dandruff (1 point), wet (1 point), eczema (2 points), bloody crusting (2 points), and skin dehydration (1 point for mild, 2 points for medium, 3 points for severe). The scores for each criterion were added together, with a maximum possible score of 10 points.

### Histological analysis

At necropsy, a skin sample measuring 1 × 1 cm was removed using scissors. The sample was placed in an embedding cassette with a sponge and fixed in 4% formaldehyde for 24 hours. After fixation, it was stored in 70% ethanol. The samples were then processed using a histomat (Leica Biosystems) and embedded in paraffin as part of the routine procedures (Jodeleit et al, 2020).

The paraffin-embedded samples were cut into 3-μm sections and stained with H&E staining using Carl Roth reagents. The stained sections were evaluated and scored on the basis of the following criteria: influx of inflammatory cells into the epidermis (scored as 1 for few, 2 for major, 3 for confluent), influx of inflammatory cells into the dermis (scored as 1 for few, 2 for major, 3 for confluent), influx of inflammatory cells into the subcutis (scored as 1 for few, 2 for major, 3 for confluent), multilayer epithelium (scored as 1 for minor, 2 for major), and ceratosis (scored as 1). The histological inflammatory skin disease score for each criterion was added together to obtain a total score ranging from 0 to 12. Representative sections were captured using an AxioVert 40 CFL camera (Zeiss) using the Zeiss ZE n2 lite software. The images were then processed using Adobe Photoshop CC, applying a tonal correction to enhance contrasts within the pictures.

### Immunohistochemistry

Tissue samples of the skin were fixed in 4% formaldehyde for 24 hours and then stored in 70% ethanol. The fixed samples were processed in a histomat (Leica Biosystems) and embedded in paraffin as part of routine procedures. The paraffin-embedded samples were cut into 3-μm sections.

For immunostaining, the sections were deparaffinized with xylene and ethanol. Antigen retrieval was performed using sodium citrate buffer in a water bath set at 58 °C. After overnight incubation, the slides were cooled in the fridge for 10–20 minutes. Subsequently, they were washed with Tris-buffered saline (TBS), and blocking buffer (1% BSA in 1 × TBS) was added for 60 minutes at room temperature.

After the removal of the blocking buffer, the first antibody was diluted in 100 μl of 1 × TBS with 1% BSA (at a dilution of 1:100) and incubated overnight at 4 °C with parafilm sealing the slides (Table 6 shows the antibodies used). After 2 washes with 1 × TBS, the second antibody was added at a concentration of 1:400 in 100 μl of blocking buffer and incubated for 60 minutes at room temperature.

The slides were then washed in 1 × TBS and sealed with cover slides with mounting medium (anti-fade gold, Thermo Fisher Scientific). Images of the stained sections were captured using an Axiokop 40 CFL camera (Zeiss). In Adobe Photoshop CC, tonal correction was applied to enhance contrasts within the images.

### Isolation of human leucocytes

Spleens of the mice were minced, and cells were filtrated through a 70-μl cell strainer. The filtered cells were then centrifuged at 1400*g* for 5 minutes and suspended in FACS buffer, which consisted of 1 × PBS, 2 mM EDTA, and 2% fetal calf serum. This process was performed to isolate human leukocytes, following the previously described methods (Jodeleit et al, 2020).

After isolation, the human leukocytes were labeled according to the specifications provided in Table 6.

### Detection of cytokines in mouse skin

Approximately 1 × 1 cm sections of shaved and depilated skin were collected and immediately frozen in dry ice. To extract proteins, 750 μl of protease inhibitor cocktail (cOmplete, Roche) was added to the frozen samples, following the manufacturer’s instructions. The samples were then milled for 10 minutes at 50 Hz using a Tissuelyser II (Qiagen) with a 5-mm stainless steel bead. After milling, 150 μl of the supernatants were collected, shock frozen, and stored at −80 °C.

To analyze the levels of human IL-4, IL-12p70, and IL-17A, a Luminex MAGPIX system (Luminex) was used. A ProcartaPlex Human High Sensitivity Basic Kit (Thermo Fisher Scientific, catalog number EPX010-10420-901) was utilized for the detection of these cytokines. The specific catalog numbers for the antibodies used are as follows: human IL4 (Thermo Fisher Scientific, catalog number EPXS010-10225-901), human IL-12p70 (Thermo Fisher Scientific, catalog number EPX01A-10238-901), and human IL-17A (Thermo Fisher Scientific, catalog number EPX01A-12017-901).

Similarly, the levels of mouse MCP3 (CCL7) and mouse TSLP were analyzed using the Luminex MAGPIX system. For this, a ProcartaPlex Mouse Basic Kit (Thermo Fisher Scientific, catalog number EPX010-20440-901) was employed. The specific catalog numbers for the antibodies used are as follows: mouse MCP3 (Thermo Fisher Scientific, catalog number EPX01A-26006-901) and mouse TSLP (Thermo Fisher Scientific, catalog number EPX01A-26095-901).

### Statistical analysis

Statistical analysis was conducted using R, a language and environment for statistical computing (R Foundation for Statistical Computing; https://www.R-project.org). The variables were summarized using mean, SD, sample size (n), difference, and 95% confidence interval. Normality of the data distribution was assessed using the Shapiro–Wilk test, and homogeneity of variances was tested using the Levene test. Because the data did not follow a normal distribution, a Wilcoxon test with continuity correction was performed. In cases where a Student’s *t*-test was used, it is explicitly mentioned in the text.

Cumming plots, which are generated using the dabest package, were employed for data presentation and comparison. Cumming plots are a novel approach to data analysis that go beyond *P*-values (Ho et al, 2019). These plots are used to analyze large samples and multiple groups. They utilize bootstrap-coupled estimation plots to present the full sampling error curve of the effect size, providing a graded representation of the distribution. The difference axis in the plot enhances the clarity of the comparison being made. Furthermore, the relative size of the confidence interval offers a specific measure of precision, separate from the magnitude, unlike *P*-values. The bootstrapping technique employed in the estimation makes the method robust and versatile. In addition, the difference diagram encourages quantitative reasoning about the system under study by focusing on effect size (Ho et al, 2019).

Heatmaps were generated in R using the heatmap.2 package. Mosaic plots were performed using the VCB package, whereas network analysis utilized the igraph package.

## Ethics Statement

All donors provided written informed consent before participating in the study. The study itself was approved by the Institutional Review Board of the Medical Faculty at the University of Munich, and the approval covered the period from 2015 to 2022.

Regarding the animal studies, they were conducted in accordance with the regulations and guidelines set forth by the animal welfare committees of the government of Upper Bavaria (Bavaria, Germany). The specific approval number for the animal studies was ROB-55.2-2532.Vet_02-19-129. Compliance with German Animal Welfare Laws was ensured throughout the duration of the studies.

## Data Availability Statement

All data are included in the Supplementary Materials.

## ORCIDs

Marietta Schindler: http://orcid.org/0009-0003-2271-3933

Paula Schuster-Winkelmann: http://orcid.org/0000-0002-6516-6912

Veronika Weß: http://orcid.org/0000-0002-5072-8767

Sophia Czell: http://orcid.org/0009-0004-2040-9361

Franziska Rueff: http://orcid.org/0000-0001-7109-8031

Andreas Wollenberg: http://orcid.org/0000-0003-0177-8722

Matthias Siebeck: http://orcid.org/0000-0001-5290-5344

Roswitha Gropp: http://orcid.org/0000-0003-4756-216X

## Conflict of Interest

The authors state no conflict of interest.

## Supplementary Material

Supplementary Figure S1 Gating strategy. (**a**) Human PBMC. (**b**) Human leukocytes isolated from mouse spleen. (**c**) Wild-type mouse spleen, unstained control. K, thousand; SSC-A, side scatter area; SCC-H, side scatter height.
