# Supplementary material for: NOD/Scid IL2Rγnull Mice Reconstituted with PBMCs from Patients with Atopic Dermatitis or Psoriasis Vulgaris Reflect the Respective Phenotype
Source: JID Innov. 2024 Feb 3;4(3):100268. doi: 10.1016/j.xjidi.2024.100268 (PMC11087984; doi:10.1016/j.xjidi.2024.100268)
Supplement: Supplementary Figure S1 — Gating strategy. (a) Human PBMC. (b) Human leukocytes isolated from mouse spleen. (c) Wild-type mouse spleen, unstained control. K, thousand; SSC-A, side scatter area; SCC-H, side scatter height. [file mmc1.pdf]

A

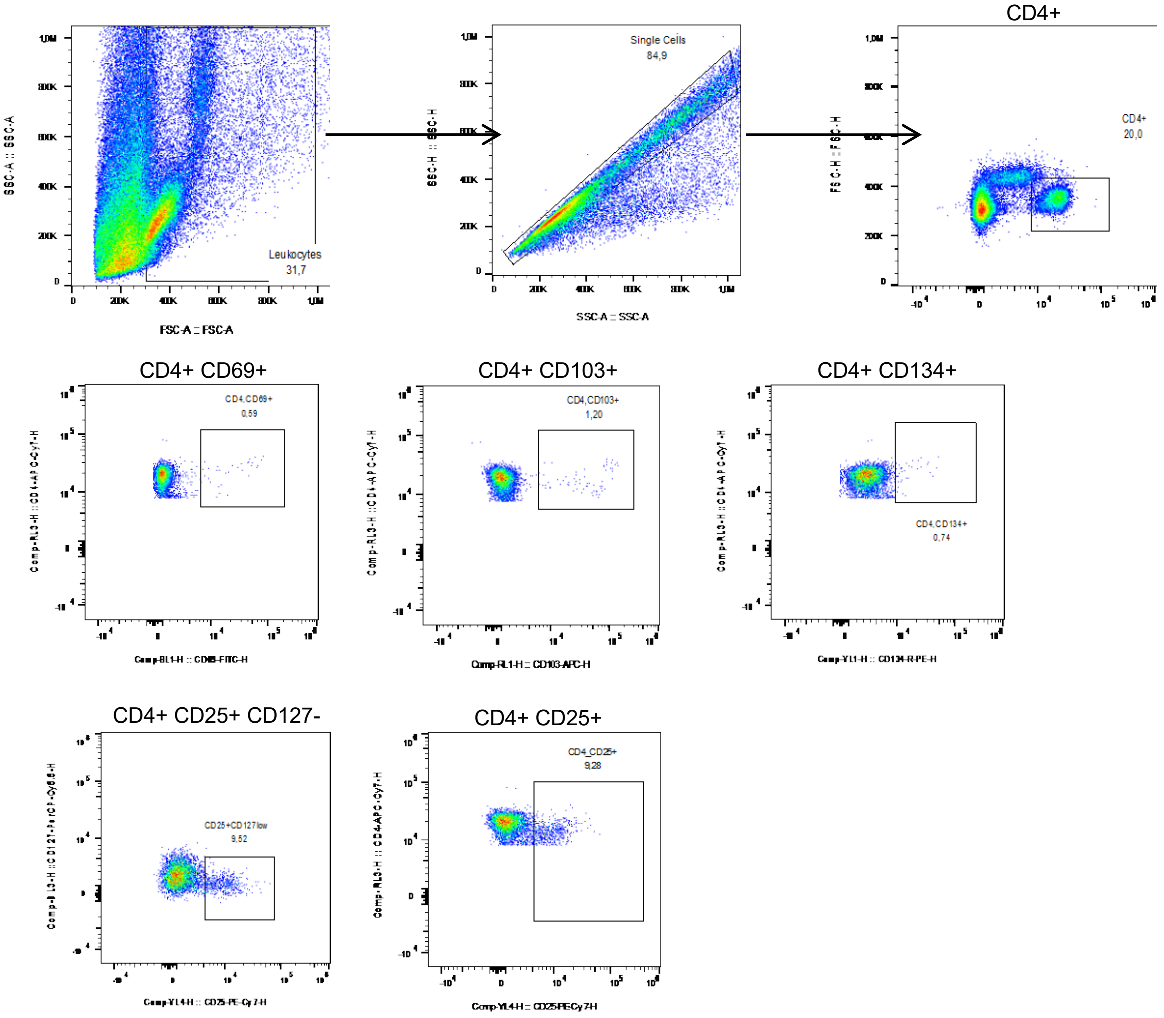
CD4+
Leukocytes
31,7
Single Cells
84,9
CD4+
20,0
FSC-A :: FSC-A
SSC-A :: SSC-A
CD4+ CD69+
CD4,CD69+
0,59
CD4+ CD103+
CD4,CD103+
1,20
CD4+ CD134+
CD4,CD134+
0,74
CD4+ CD25+ CD127-
CD25+CD127low
9,52
CD4+ CD25+
CD4_CD25+
9,28

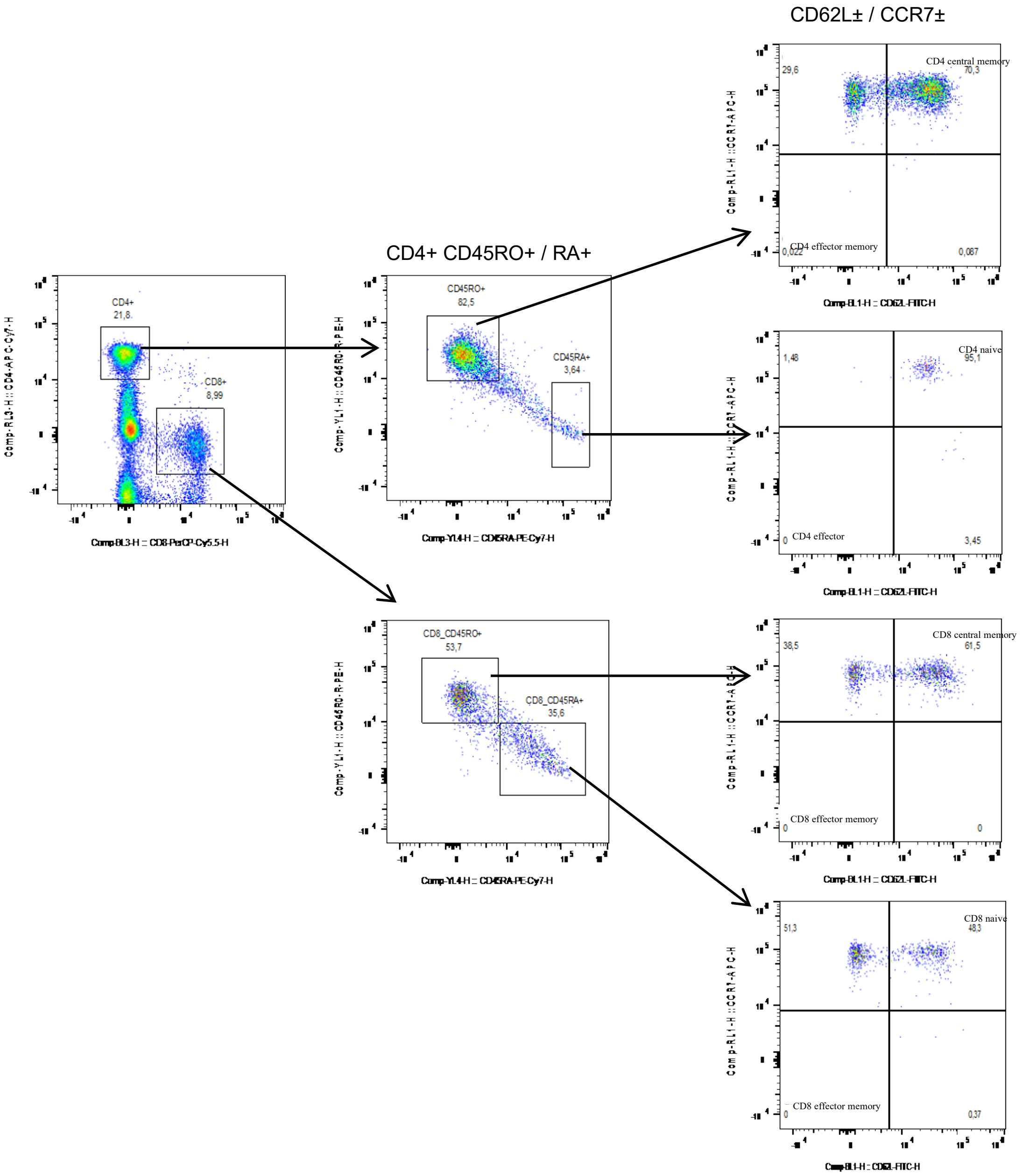
CD62L± / CCR7±
CD4+ CD45RO+ / RA+
CD4+
21,8
CD8+
8,99
Comp-BL3-H :: CD8-PerCP-Cy5.5-H
Comp-RL3-H :: CD4-APC-Cy7-H
CD45RO+
82,5
CD45RA+
3,64
Comp-YL4-H :: CD45RA-PE-Cy7-H
Comp-YL1-H :: CD45RO-R-PE-H
CD8_CD45RO+
53,7
CD8_CD45RA+
35,6
CD4 central memory
70,3
29,6
CD4 effector memory
0,022
0,087
CD4 naive
95,1
1,48
CD4 effector
0
3,45
CD8 central memory
61,5
38,5
CD8 effector memory
0
0
CD8 naive
48,3
51,3
CD8 effector memory
0
0,37
Comp-BL1-H :: CD62L-FITC-H
Comp-RL1-H :: CCR7-APC-H

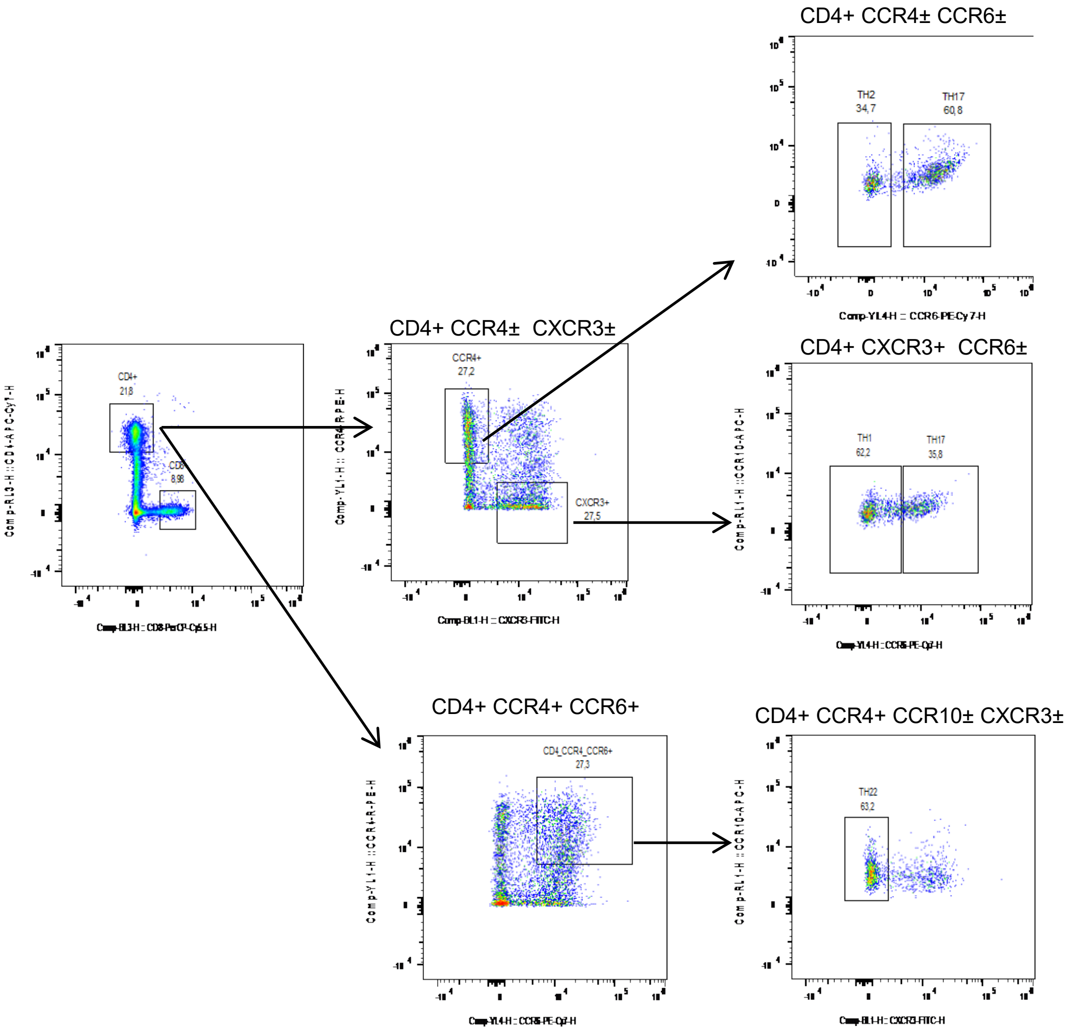

CD4+ CCR4± CCR6±
TH2
34,7
TH17
60,8
Comp-YL4-H :: CCR6-PE-Cy7-H
CD4+ CCR4± CXCR3±
CCR4+
27,2
CXCR3+
27,5
Comp-BL1-H :: CXCR3-FITC-H
CD4+
21,8
Comp-BL3-H :: CD8-PerCP-Cy5.5-H
Comp-RL3-H :: CD4-APC-Cy7-H
CD4+ CXCR3+ CCR6±
TH1
62,2
TH17
35,8
Comp-RL1-H :: CCR10-APC-H
CD4+ CCR4+ CCR6+
CD4_CCR4_CCR6+
27,3
Comp-YL1-H :: CCR4-R-PE-H
CD4+ CCR4+ CCR10± CXCR3±
TH22
63,2
Comp-BL1-H :: CXCR3-FITC-H

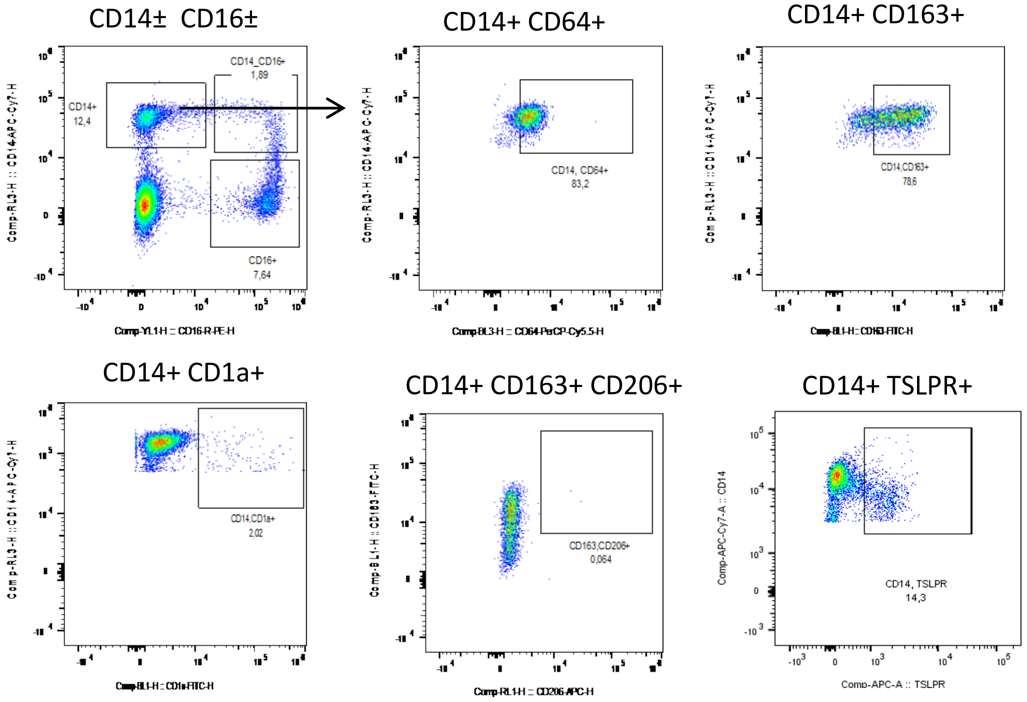
CD14± CD16±
CD14+
12,4
CD14_CD16+
1,89
CD16+
7,64
Comp-YL1-H :: CD16-R-PE-H
CD14+ CD64+
CD14, CD64+
83,2
Comp-BL3-H :: CD64-PerCP-Cy5.5-H
CD14+ CD163+
CD14,CD163+
78,6
CD14+ CD1a+
CD14,CD1a+
2,02
CD14+ CD163+ CD206+
CD163,CD206+
0,064
Comp-RL1-H :: CD206-APC-H
CD14+ TSLPR+
CD14, TSLPR
14,3
Comp-APC-A :: TSLPR
Comp-APC-Cy7-A :: CD14

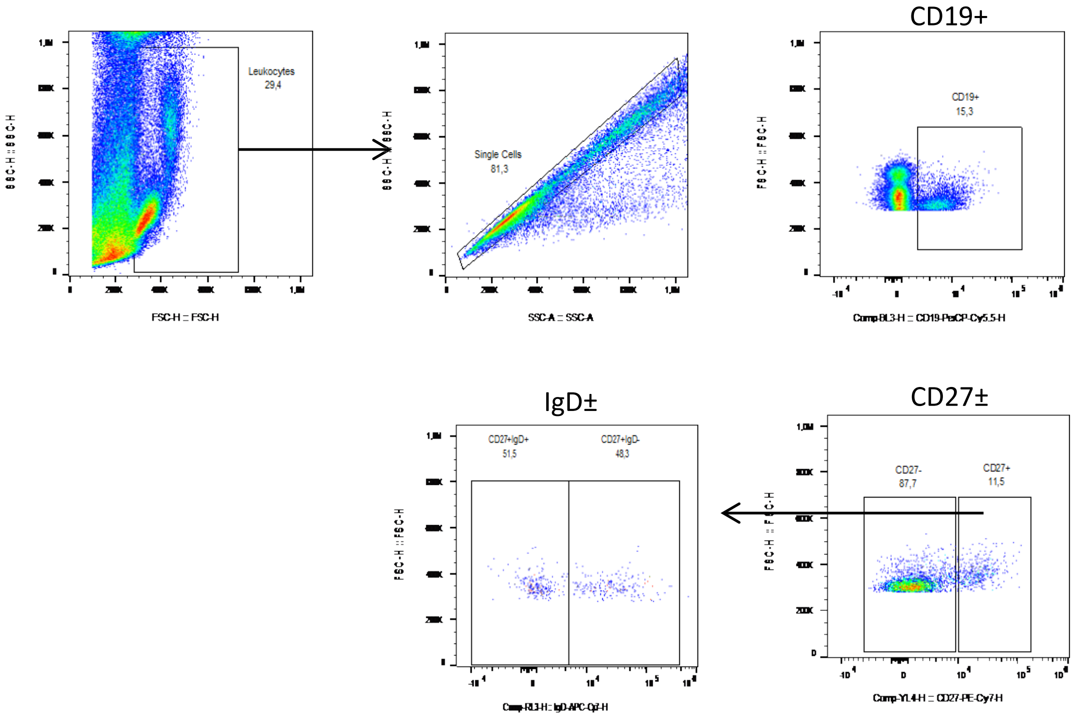
Leukocytes
29,4
FSC-H :: FSC-H
Single Cells
81,3
SSC-A :: SSC-A
CD19+
CD19+
15,3
Comp-BL3-H :: CD19-PerCP-Cy5.5-H
IgD±
CD27+IgD+
51,5
CD27+IgD-
48,3
CD27±
CD27-
87,7
CD27+
11,5
Comp-YL4-H :: CD27-PE-Cy7-H

B

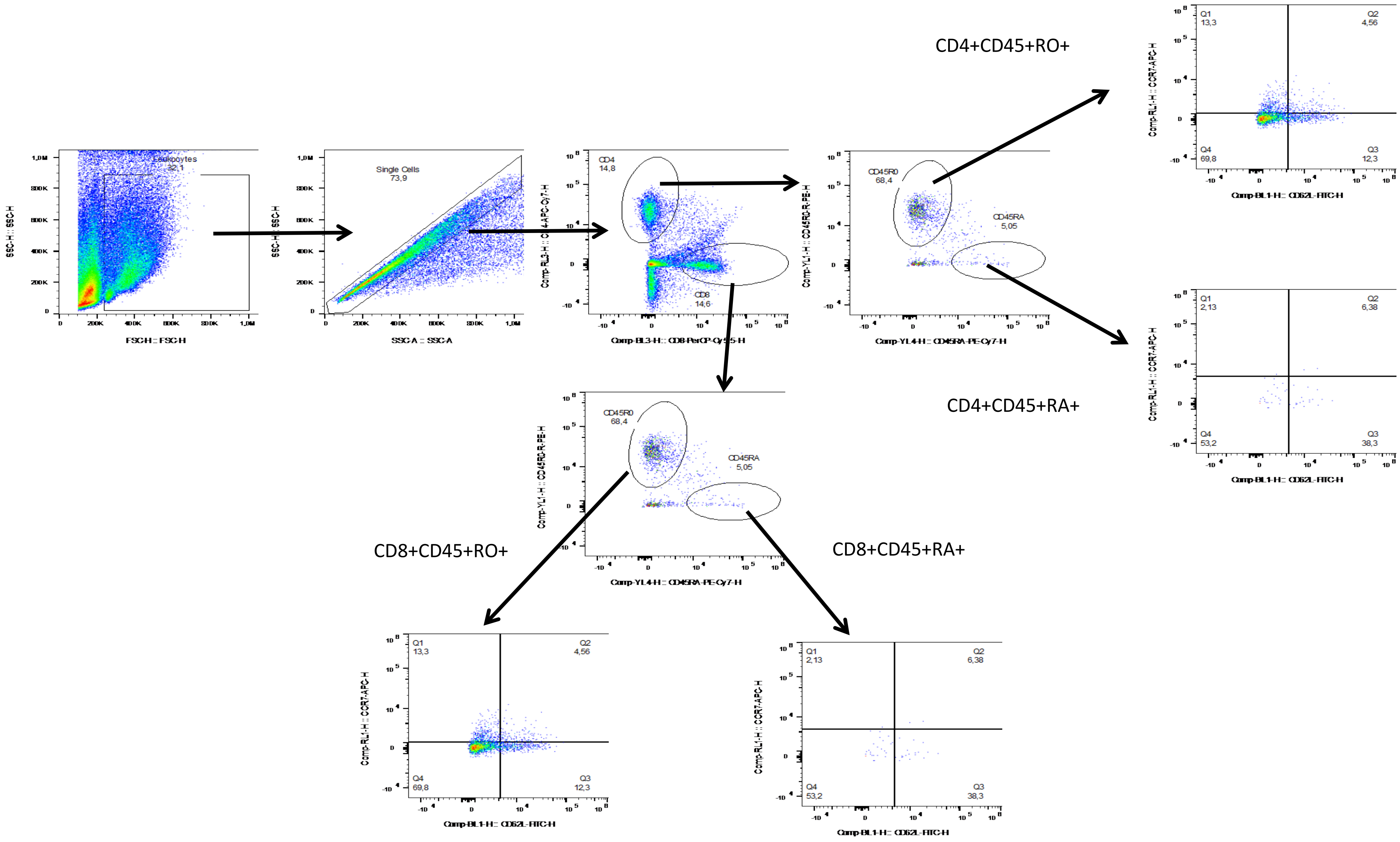
Leukocytes
32,1
FSC-H :: FSC-H
SSC-H :: SSC-H
Single Cells
73,9
SSC-A :: SSC-A
CD4
14,8
CD8
14,6
CD45R0
68,4
CD45RA
5,05
Comp-YL4-H :: CD45RA-PE-Cy7-H
Comp-YL1-H :: CD45RO-R-PE-H
CD4+CD45+RO+
CD4+CD45+RA+
CD8+CD45+RO+
CD8+CD45+RA+
Q1
13,3
Q2
4,56
Q3
12,3
Q4
69,8
Q1
2,13
Q2
6,38
Q3
38,3
Q4
53,2
Comp-BL1-H :: CD62L-FITC-H
Comp-RL1-H :: CCR7-APC-H

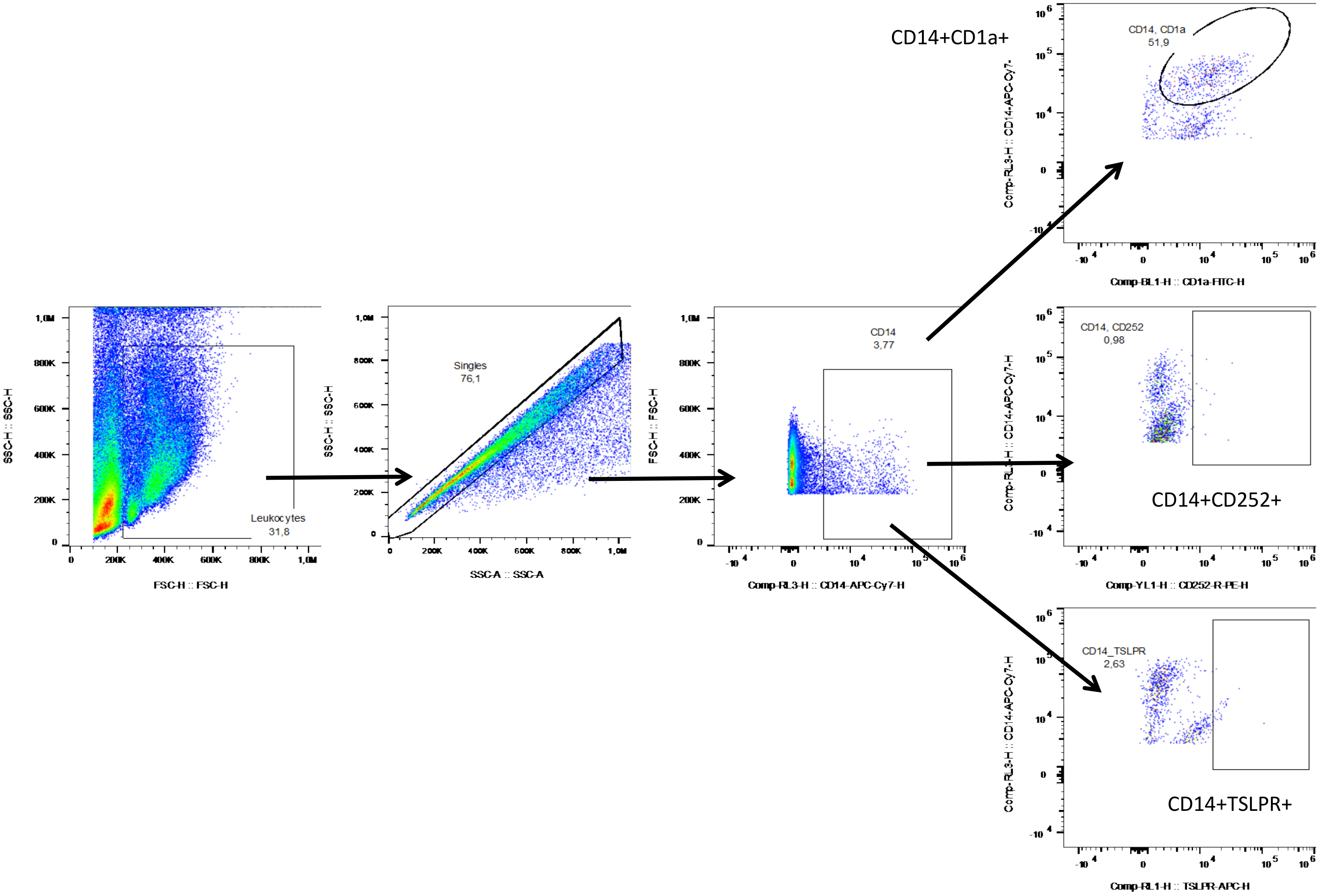
Leukocytes
31,8
FSC-H :: FSC-H
SSC-H :: SSC-H
Singles
76,1
SSC-A :: SSC-A
CD14
3,77
Comp-RL3-H :: CD14-APC-Cy7-H
FSC-H :: FSC-H
CD14+CD1a+
CD14, CD1a
51,9
Comp-BL1-H :: CD1a-FITC-H
CD14, CD252
0,98
CD14+CD252+
Comp-YL1-H :: CD252-R-PE-H
CD14_TSLPR
2,63
CD14+TSLPR+
Comp-RL1-H :: TSLPR-APC-H

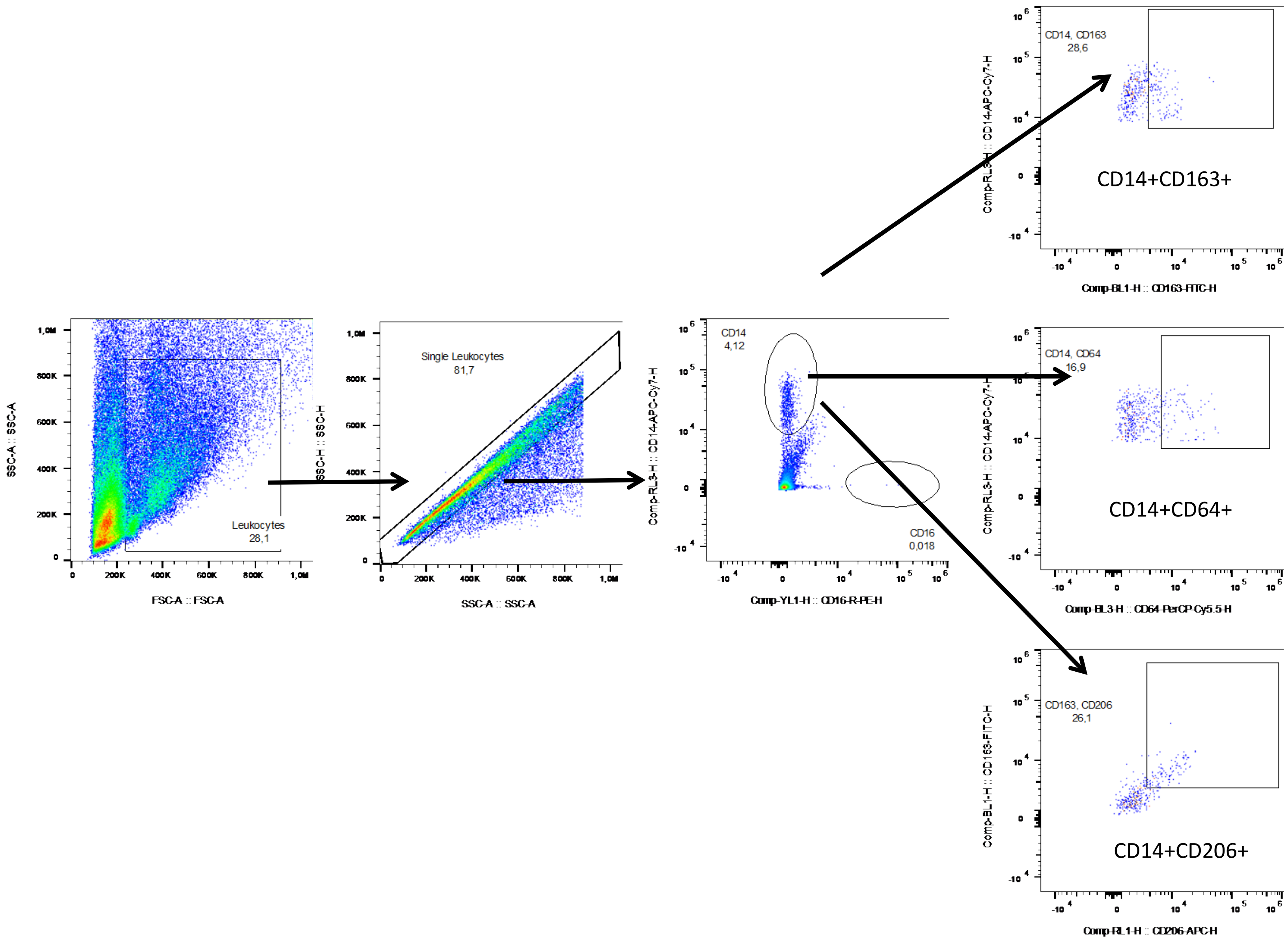

Leukocytes
28,1
SSC-A :: SSC-A
FSC-A :: FSC-A
Single Leukocytes
81,7
SSC-H :: SSC-H
SSC-A :: SSC-A
CD14
4,12
CD16
0,018
Comp-RL3-H :: CD14-APC-Cy7-H
Comp-YL1-H :: CD16-R-PE-H
CD14, CD163
28,6
CD14+CD163+
Comp-BL1-H :: CD163-FITC-H
CD14, CD64
16,9
CD14+CD64+
Comp-BL3-H :: CD64-PerCP-Cy5.5-H
CD163, CD206
26,1
CD14+CD206+
Comp-BL1-H :: CD163-FITC-H
Comp-RL1-H :: CD206-APC-H

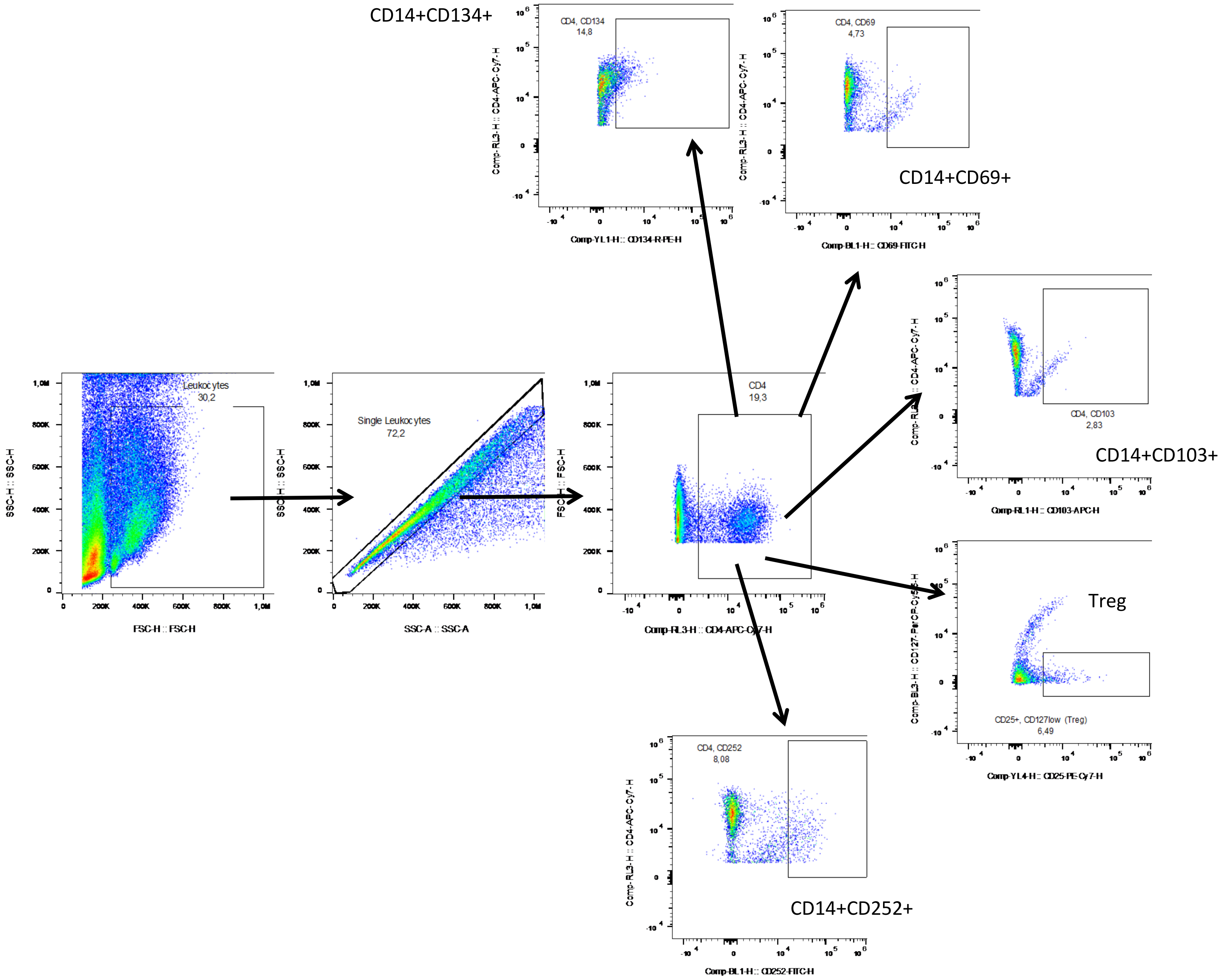

CD14+CD134+
CD4, CD134
14,8
Comp-RL3-H :: CD4-APC-Cy7-H
Comp-YL1-H :: CD134-R-PE-H
CD4, CD69
4,73
CD14+CD69+
Comp-BL1-H :: CD69-FITC-H
Leukocytes
30,2
SSC-H :: SSC-H
FSC-H :: FSC-H
Single Leukocytes
72,2
SSC-A :: SSC-A
CD4
19,3
FSC-H :: FSC-H
Comp-RL3-H :: CD4-APC-Cy7-H
CD4, CD103
2,83
CD14+CD103+
Comp-RL1-H :: CD103-APC-H
Treg
CD25+, CD127low (Treg)
6,49
Comp-BL3-H :: CD127-PerCP-Cy5.5-H
Comp-YL4-H :: CD25-PE-Cy7-H
CD4, CD252
8,08
CD14+CD252+
Comp-BL1-H :: CD252-FITC-H

C

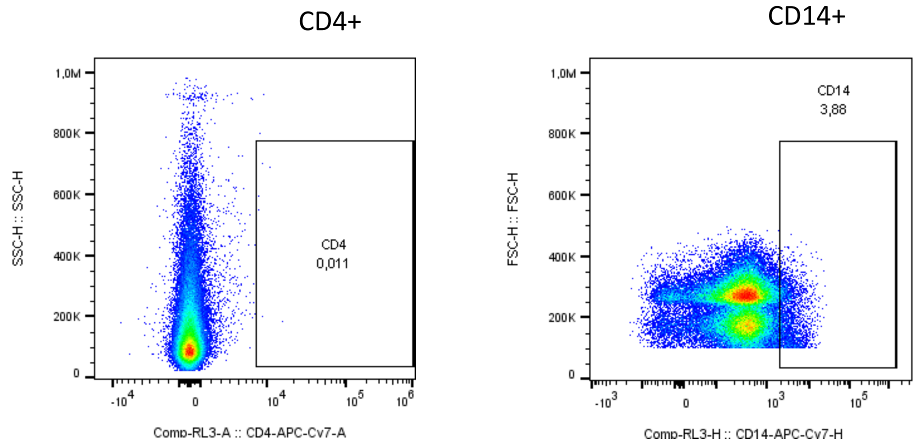

Supplementary Figure S1 Gating Strategy. (A) Human PBMC; (B) Human leukocytes isolated from mouse spleen; (C) Wild type mouse spleen, unstained control.
